# Effects of paternal arachidonic acid supplementation on offspring behavior and hypothalamus inflammation markers in the mouse

**DOI:** 10.1371/journal.pone.0300141

**Published:** 2024-03-21

**Authors:** Atenea Vázquez-Sánchez, Dalia Rodríguez-Ríos, Dannia Colín-Castelán, Jorge Molina-Torres, Enrique Ramírez-Chávez, Gloria del Carmen Romo-Morales, Silvio Zaina, Gertrud Lund

**Affiliations:** 1 Department of Genetic Engineering, CINVESTAV Irapuato Unit, Irapuato, Mexico; 2 Division of Health Sciences, Department of Medical Sciences, University of Guanajuato, Leon Campus, Leon, Gto., Mexico; 3 Department of Biotechnology and Biochemistry, CINVESTAV Irapuato Unit, Irapuato, Mexico; Universitatsklinikum Wurzburg, GERMANY

## Abstract

Arachidonic acid (AA) is involved in inflammation and plays a role in growth and brain development in infants. We previously showed that exposure of mouse sires to AA for three consecutive generations induces a cumulative change in fatty acid (FA) involved in inflammation and an increase in body and liver weight in the offspring. Here, we tested the hypothesis that paternal AA exposure changes the progeny’s behavioral response to a proinflammatory insult, and asked whether tissue-specific FA are associated with that response. Male BALB/c mice were supplemented daily with three doses of AA for 10 days and crossed to non-supplemented females (n = 3/dose). Two-month-old unsupplemented male and female offspring (n = 6/paternal AA dose) were exposed to Gram-negative bacteria-derived lipopolysaccharides (LPS) or saline control two hours prior to open field test (OFT) behavioral analysis and subsequent sacrifice. We probed for significant effects of paternal AA exposure on: OFT behaviors; individual FA content of blood, hypothalamus and hypothalamus-free brain; hypothalamic expression profile of genes related to inflammation (*Tnfa*, *Il1b*, *Cox1*, *Cox2*) and FA synthesis (*Scd1*, *Elovl6*). All parameters were affected by paternal AA supplementation in a sex-specific manner. Paternal AA primed the progeny for behavior associated with increased anxiety, with a marked sex dimorphism: high AA doses acted as surrogate of LPS in males, realigning a number of OFT behaviors that in females were differential between saline and LPS groups. Progeny hypothalamic *Scd1*, a FA metabolism enzyme with documented pro-inflammatory activity, showed a similar pattern of differential expression between saline and LPS groups at high paternal AA dose in females, that was blunted in males. Progeny FA generally were not affected by LPS, but displayed non-linear associations with paternal AA doses. In conclusion, we document that paternal exposure to AA exerts long-term behavioral and biochemical effects in the progeny in a sex-specific manner.

## Introduction

Arachidonic acid (AA) is a 20-carbon chain omega-6 polyunsaturated fatty acid (PUFA). AA can be synthesized endogenously from linoleic acid, an essential FA in humans, or obtained directly from the diet. In adults, the average daily intake of AA in developed countries is 100–250 mg, mainly obtained from eggs, chicken and fish such as sardines and salmon [1]. Breastmilk represents an important source of dietary AA for the developing infants providing an estimated average intake of 118–178 mg/day and infant formulas are typically adjusted to mimic breastfed AA levels [2].

AA is primarily in membrane phospholipids of brain, liver, skeletal muscle, platelets, and immune cells and its biological properties complex [3]. On the one hand, AA is the precursor of pro-inflammatory factors such as prostaglandins, leukotrienes, and thromboxane that are generated via the cyclooxygenase, lipoxygenase, and cytochrome P450 pathways [4, 5]. Dysregulations in these pathways play important roles in diseases associated with aberrant immune function such as cardiovascular disease, cancer, allergies and mood disorders. On the other hand, strong evidence exists for a pivotal, beneficial role of AA in driving physiological development (reviewed in [6]). AA accumulates in the fetal circulation and brain, and the placenta in animal models and humans, suggesting an evolutionarily conserved role in supporting the function of those critical organs in mammals [7–9]. Another example are platelets, where AA can represent up to 25% of total fatty acid (FA) [10]. Notably, those studies indicate that organ enrichment in AA is driven mainly by dietary intake, rather than by endogenous synthesis. Critically, dietary AA supplementation does not increase its inflammation-related derivatives, implying that AA supports inflammation as part of the physiological response to injury or disease, rather than being pro-inflammatory *per se* [11].

The aforementioned data support a role for AA in early organ development, in particular neural and immune systems. Indeed, AA, in addition to docosahexaenoic acid (DHA, 22:6n-3) constitute up to 40% of total brain FA [12] and dietary intervention studies including both FA have been associated with positive cognitive outcomes [13]. A systematic review of AA supplementation in adults found no adverse effects of AA intake <1500mg/day on immune function, while one study showed improved cognitive function following a 1-month dietary intervention with AA enriched oil in elderly men [14, 15]. Furthermore, a recent study in young pigs found that exposure to an AA supplement led to higher activity levels and less time spent asleep compared to control fed pigs [16].

We have recently shown that a consecutive three generation supplementation in mouse (females during pregnancy or in males 10 days prior to coitus), is associated with a cumulative increase in offspring whole body weight (BW) and liver weight [17]. In addition, we found that AA parental exposure led to a cumulative increase in offspring liver anti-inflammatory FA cis-7-hexadecenoic acid (16:1n-9; HDA), which had been previously shown to specifically accumulate in lipid droplets of monocytes challenged with AA [18]. The observed increase in BW—if associated with increased availability of cellular energy sources—and the presence of anti-inflammatory liver FA support the idea that parental AA exposure across generations may improve the progeny’s response to pro-inflammatory challenges. To test that hypothesis, we exploited a murine model of paternal supplementation of dietary AA, followed by a pro-inflammatory challenge in the unsupplemented progeny. Given the abundance of AA in brain tissue, and its role in both inflammation and neural development, we focused on the effects of AA on mouse behavior and brain FA, in particular the hypothalamus. The rationale for surveying the hypothalamus is its important role in regulating energy homeostasis and inflammation in response to dietary FA [19]. Furthermore, we chose to explore the effects of AA along the paternal line since any effects on progeny are purely intergenerational. Additionally, most intergenerational studies have focused on the mother-fetus dyad, whereas intergenerational inheritance along the paternal line is relatively understudied [20]. We discuss the data in the context of current knowledge of intergenerational memory of paternal exposure to dietary components and inflammation.

## Materials and methods

### Mouse supplementation

**Fig 1:**
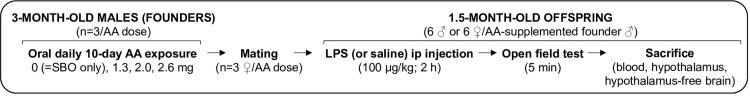
Schematic view of the animal procedures. To maintain the amount of calories constant across AA doses, each supplemented volume was adjusted to 5 μl with Nutrioli^®^, a commercial soybean oil (SBO) with no detectable levels of AA; control mice received 5 μl Nutrioli^®^ only. LPS, lipopolysaccharide; ip, intraperitoneal.

A schematic view of the animal procedures is shown in **Fig 1**. The protocol was revised and approved by the Institutional Committee for Ethics in Research of the University of Guanajuato, Mexico (authorization no. CIBIUG-A37-2018). Male BALB/c mice were bred in the Department of Pharmacy, University of Guanajuato, Guanajuato, Mexico, and subsequently housed in the animal facilities of the Division of Health Sciences, University of Guanajuato, Leon, Mexico, with *ad libitum* access to food (Harlan Laboratories 2018 standard diet) and water. Following a one-week adaptation period, three-month-old mice (founders; n = 3/AA dose) were supplemented orally for 10 consecutive days with 1.3, 2.0 and 2.6 mg (or 37.0, 55.0 or 74.0 mg/kg/day) of 98.5% pure AA (Sigma-Aldrich, no. A3611). The rationale for the choice of AA doses was manifold: 1) four points (including the zero, SBO-alone dose) were deemed sufficient to reveal any AA dose-dependent effects; 2) the values correspond to 1, 1.5 and 2 times the dose used in our previous study on intergenerational effects of supplemented AA and therefore represent an evenly spaced range of values [17]; 3) the dose range is compatible with the corresponding values observed in real-world human consumption [1]. To maintain an equal amount of calories across increasing AA doses, the volume of the supplement was adjusted to 5 μl with Nutrioli^®^, a commercial soybean oil (SBO) that has no detectable levels of AA [17]; control mice received 5 μl vector only. The physiological AA precursor linoleic acid is present in soybean oil at the concentration of 7.5 g per serving (15.4 ml) (www.nutrioli.com). This is equivalent to a maximum of 2.4 mg per 5 μl supplement and may impact on the final endogenous concentration of AA. Nonetheless, that contribution is likely limited, as supplemented linoleic acid contributes to tissue AA one order of magnitude less than supplemented AA (reviewed in [6]). Likewise, increased levels of dietary linoleic acid are not associated with increases in tissue AA content [21]. To minimize oxidation, upon vial opening AA was immediately dissolved in SBO that contains 0.01% of the antioxidant tert-butylhydroquinone. Five μl AA+SBO aliquots were stored at −20°C until use. Oral supplementation was carried out as previously described [17]. Briefly, the supplement was pipetted into the side of the mouth using a pipette with a blunted tip to avoid injury. The oral uptake was complete, as oils are highly palatable to mice. BW and food intake were measured daily during the 10-day supplementation period. Subsequently, males were crossed with non-supplemented females (1 male with 3 females per cage). The presence of a vaginal plug was used as an indication of successful mating and litter size was registered at birth. Offspring groups of each founder AA/SBO ratio (vol/vol) will be referred to as AA/SBO 0.00, 0.37, 0.68 and 1.17, corresponding to SBO only, and increasing amounts of AA, respectively. AA/SBO will be used to represent AA doses throughout the text.

### Offspring analysis

Two-month-old offspring were analyzed as follows: 1) 12 male and 12 female mice were randomly selected from litters of each AA/SBO-exposed founder and weighed (n = 24 mice per AA/SBO ratio, representing the three founders equally); 2) half of these (n = 12; 6 males and 6 females) were randomly selected and injected intraperitoneally (ip) with 100 μg/ml LPS (Sigma-Aldrich, no. L2630) dissolved in 0.9% sterile NaCl (saline); control mice received saline only. To eliminate the founder as confounding variable, the progeny equally represented each of the three founders subjected to any AA/SBO. After 90 min mice were transferred to a separate room, acclimatized for 30 min and subsequently subjected to a 5 min OFT. LPS dose and interval between ip LPS injection and OFT was as previously described [22].

OFT was performed under natural light conditions using a square transparent acrylic box (55x55 cm) with a floor divided into 9 equal squares positioned into a 3x3 matrix. All mice were tested during the first half of the light phase of their light/dark cycle i.e., between 10 am and 4 pm. The test was initiated by placing a single mouse in the middle square of the floor and letting it move freely for 5 min. Mouse behavior was recorded continuously with a video camera placed over the box. Each video session was subsequently analyzed using OBSERVER 2.0 and ANY-maze software. For the latter, we considered the middle square as the center and the 4 vertex squares as corners. The test box was carefully cleaned with alcohol and rinsed with water between consecutive OFT.

### Tissue dissection

Immediately following the OFT, offspring were sacrificed by decapitation under anesthesia with isoflurane and total blood, hypothalamus and the remaining hypothalamus-free (HF) brain were harvested in DNA/RNA Shield (Zymo Research, no. R1100-50) and stored at −80°C until further use. In the subsequent experiments, care was taken to equally represent the whole brain in each case.

### FA determination

Individual blood, hypothalamus and HF brain FA profiles were determined as FA methyl esters as previously described [17]. In the case of the hypothalamus, FA profiles were determined of tissue pooled from 6 mice (i.e., 3 male and 3 female mice per AA/SBO founder ratio). A total of 18, 16 and 13 individual FA were detected in blood, HF-brain and hypothalamus, respectively.

### RT-PCR analysis

RNA extraction and semiquantitative PCR were performed as previously described [17]. Expression of *Interleukin 1 beta* (*Il1b*) and cyclooxygenases *Cox1* and *Cox2*, was analyzed using previously reported primer pairs [22]. Primer pairs for *Tumor necrosis factor alpha* (*Tnfa*), *Stearoyl-CoA desaturase* (*Scd1*) and *Elongation of very-long-chain fatty acid 6* (*Elovl6*) are available upon request. Hypothalamic gene expression profiles were performed on three individual male or female offspring of each founder AA/ABO. Original gel images are in **S1 Raw images**.

### Statistics

Tests were performed with the following two independent variables: the AA dose (AA/SBO; quantitative discrete variable); and exposure to LPS (LPS-exposed or saline-exposed; categorical variable). Dependent variables were the BW, OFT outcome, FA, or inflammation-related factors, depending on the specific analysis. To assess the independent effects and interaction between the AA/SBO and OFT, we used two-way analysis of variance (ANOVA) followed by Scheffé’s *post hoc* test. The overall nominal p of the ANOVA was corrected for repeated measurements (Bonferroni correction) when appropriate. The *t*-test was used in selected cases. Analyses were performed on individual values of offspring (n = 6 male or female offspring) derived from each biological replicate of AA/SBO exposed males (n = 3 for each AA/SBO). Pearson’s correlation was used in the case of pooled values of hypothalamic FA content.

## Results

Our experimental protocol was designed to test the effects of paternal (founder) supplemented with increasing concentrations of AA on unsupplemented offspring traits following exposure to LPS or saline only. We supplemented mouse sires with three doses of AA for 10 days prior to coitus and sought any association of paternal AA dose with behavior open field test (OFT) outcome and inflammatory markers in non-supplemented progeny challenged with lipopolysaccharide (LPS), which mimics a Gram-negative bacteria infection. OFT outcome reveals any response in terms of anxiety or fear [23]. We included both OFT and inflammation-related factors as outcomes, as anxiety and inflammation are compounded responses to LPS [24]. The analyzed offspring traits were BW, OFT behavior, peripheral blood, hypothalamus and HF brain FA, and gene expression in the hypothalamus.

### Effects of AA supplementation on BW of exposed founder mice and their offspring

First, we asked whether increasing founder AA/SBO affected the BW of founder males or their offspring. However, neither founder nor female or male offspring showed significant differences in BW across AA/SBO groups (p = 0.833, p = 0.110 and p = 0.289, respectively, ANOVA). Furthermore, founder and offspring (female or male) BW did not significantly correlate with founder AA/SBO (r = 0.132, p = 0.682; r = -0.573, p = 0.066; and r = -0.572, p = 0.066, respectively) (**S1 Fig**). In accordance with the lack of association between founder BW and AA/SBO, food intake was not significantly different across founder AA/SBO groups (p = 0.489). Likewise, the number of pups (female and male) derived from founder males, did not differ significantly across treatment groups (p = 0.270 and p = 0.296, respectively) and showed no correlation with founder AA/SBO (r = -0.049, p = 0.907), or founder or offspring BW (r = 0.361, p = 0.249 and r = -0.214, p = 0.505, respectively).

### Interactions between founder AA/SBO treatment and offspring behavior outcome following LPS exposure

Following the OFT, we successfully registered a total of 30 ANY-maze software-analyzed behaviors such as distance, line crossings, speed, and freezing (**S1 Table**). The statistical criteria for identifying founder AA/SBO-dependent effects on offspring OFT behavior was significant difference across founder AA/SBO groups and/or LPS exposure (ANOVA; Bonferroni corrected threshold p = 1.7x10^-3^—*i*.*e*. nominal p divided by the number or detected behaviors—and Scheffé’s *post hoc* p<0.05). Analyses were performed on female and male offspring separately (n = 6 each) and separate tests were run in LPS-exposed or saline-exposed offspring.

**Fig 2:**
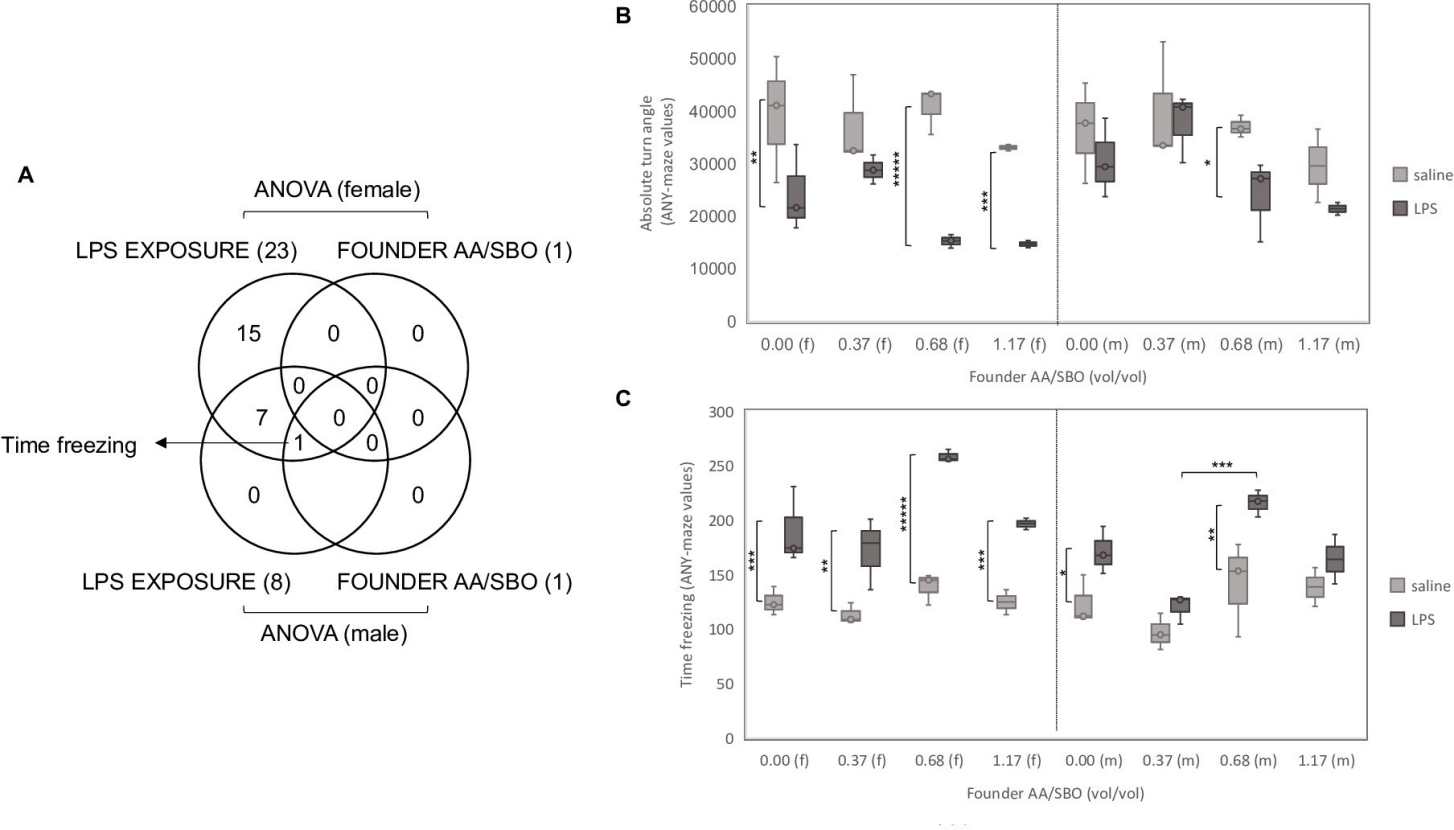
Female and male OFT behaviors associated with LPS exposure and/or founder AA/SBO. A) Venn diagrams of significant OFT behaviors in female and male progeny (ANOVA, Bonferroni adjusted p<0.00167); B) Representative OFT behavior that responded more significantly to LPS exposure in female compared to male offspring; C) OFT behavior that differed across AA/SBO 1.17 in LPS-exposed males; f, females, m, males; *,**,***;****,*****, p<0.05, 0.01, 0.001, 10^−4^ and 10^−5^, respectively (Scheffé’s *post hoc* and t-test; solid and dotted brackets, respectively).

**Fig 3:**
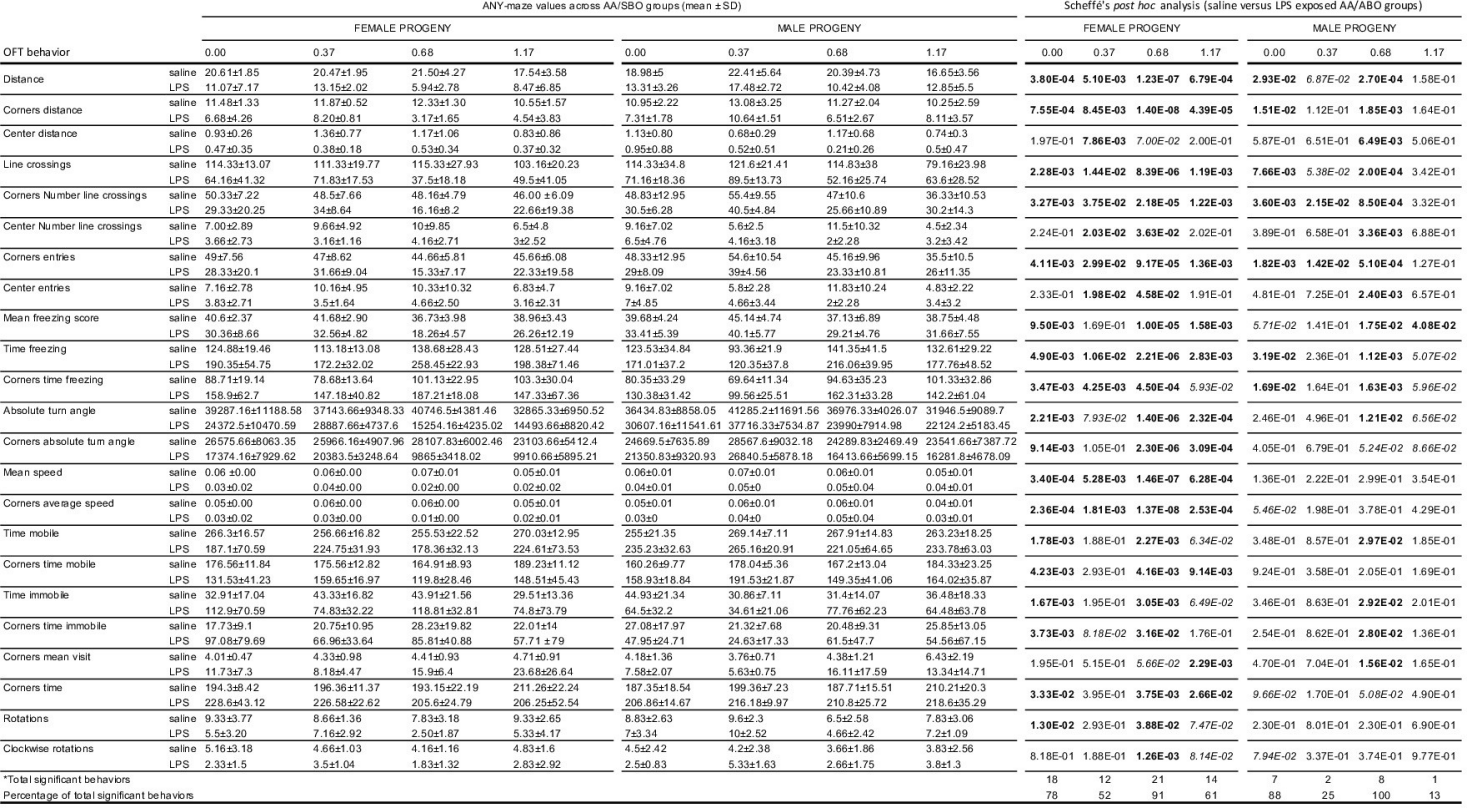
Scheffé’s post hoc analysis of progeny OFT behaviors that differed across saline-exposed and LPS-exposed AA/SBO groups. *OFT behaviors that showed significance following ANOVA (Bonferroni adjusted p<0.00167) and Scheffé’s post hoc analysis (p<0.05, in bold); mean, n = 6/experimental group.

**Fig 4:**
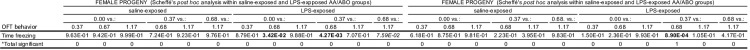
Scheffé’s post hoc analysis of progeny OFT behaviors affected by founder AA/SBO treatment. *OFT behaviour that showed significance following ANOVA (Bonferroni adjusted p<0.00167) and Scheffé’s post hoc analysis (p<0.05, in bold).

We first compared OFT behaviors across and within LPS-exposed and saline-exposed groups. As expected, several OFT behaviors differed significantly between saline-exposed and LPS-exposed groups. However, sexual dimorphism was strong, as significantly affected behaviors were ~three-fold more abundant in females compared to males (23 and 8, respectively) (**S2 Table and Fig 2A**). Conversely, only one OFT behavior differed significantly across founder AA/SBO groups, i.e., “time freezing” in male progeny **(S2 Table**). All significant male OFT behaviors were also significant in female progeny and in either case consistent with elevated anxiety-like behavior, *i*.*e*., increased time spent in corners and a reduction in overall movement with increasing founder AA/SBO (**Figs 2B and 2C and 3**). Furthermore, ranking AA/SBO ratios by number of OFT behaviors that were significant in the *post hoc* analysis, most differences were found in the AA/SBO 0.68 group in both female and male progeny; i.e. 91% (21/23) and 100%, respectively (**Figs 3** and **4**). Notably, in AA/SBO 1.17 progeny, 13 and 61% of male and female OFT behaviors, respectively, differed between saline-exposed and LPS-exposed progeny. To understand whether the lack of response to LPS in males represented a blunted response or an enhanced response to saline exposure, we first probed for correlations between male and female behaviors in either condition. Female and males OFT behaviors were more strongly correlated in the LPS-exposed compared to the saline-exposed group, suggesting that (**S3 Table**). To explain that observation, we carried comparisons females and males of each AA/SBO, for the 14 behaviors that correlated between male and female progeny. The analysis revealed that most significant differences were in the AA/SBO 1.17 of the saline-exposed group (**S3 Table**). The data indicate that paternal AA/SBO 1.17 blunts the difference between saline and LPS in males, essentially acting a surrogate for LPS in the progeny. **Fig 2B and 2C** offers an example of that trend in two representative OCT behaviors among the 14 behaviors that correlated between male and female progeny: female and male "absolute turn angle" and "time freezing" realign at high AA/SBO doses.

### Interactions between LPS exposure and paternal AA treatment in offspring FA

A previous analysis from our laboratory showed that AA treatment for three generations led to cumulative changes in liver FA content, suggesting that founder AA/SBO may affect progeny FA in multiple tissues in our model [17]. Since founder AA/SBO interacted with the outcome of progeny exposure to LPS and OFT behaviors, we reasoned that changes in the FA pool may be associated with the two latter variables. Therefore, we first asked whether offspring FA of peripheral blood, hypothalamus and HF-brain were associated with founder AA/SBO. The statistical criteria for identifying founder AA/SBO-dependent effects on offspring FA was as described for OFT behavior. Due to limiting amount of tissue, FA content was analyzed of hypothalami pooled from three male and three female mice. Therefore, statistical tests of founder AA/SBO effects on hypothalamic FA content were restricted to paired *t*-test and Pearson’s correlation.

**Fig 5:**
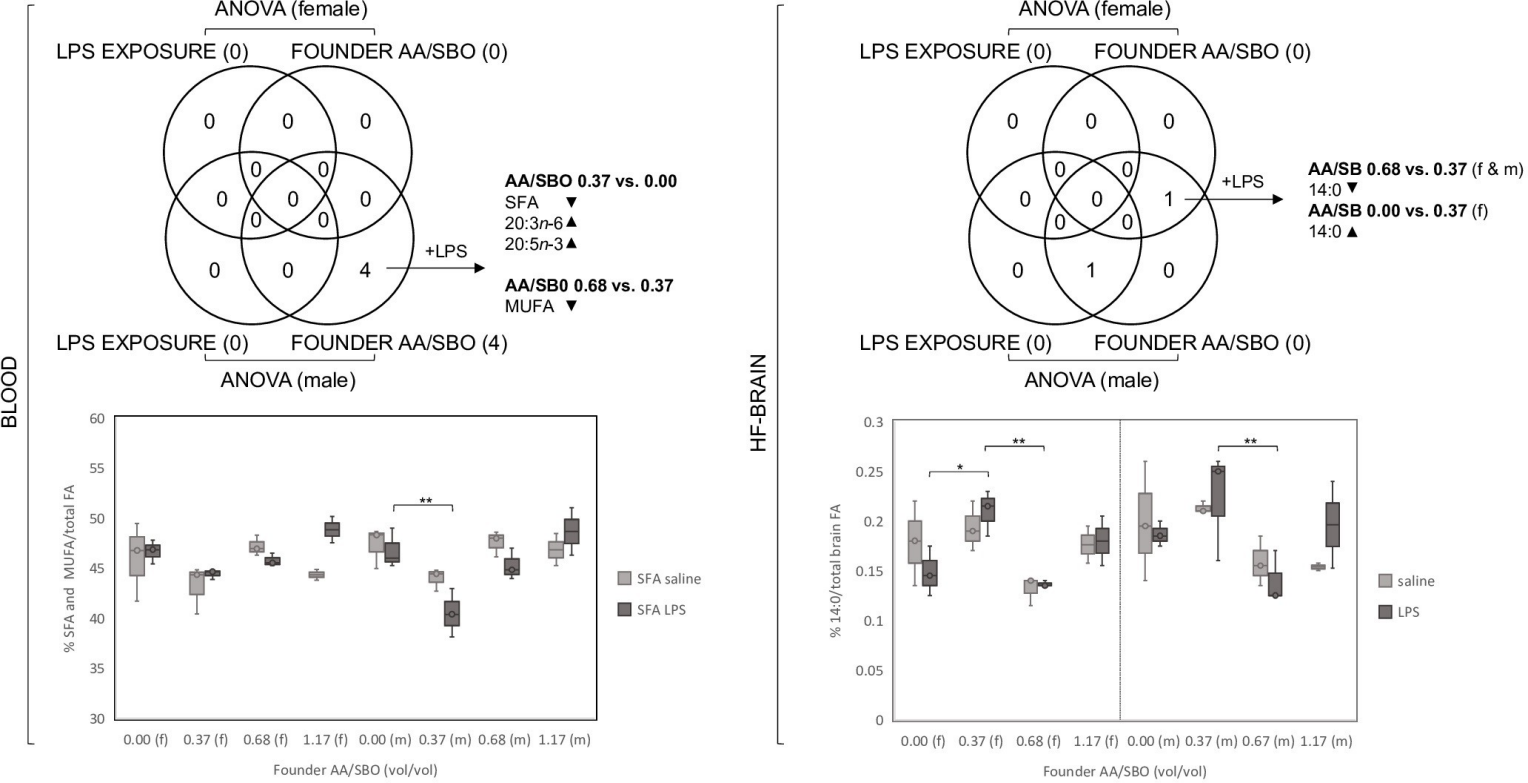
Female and male blood and HF-brain FA that were associated with LPS exposure and/or founder AA/SBO. Venn diagrams of significant FA in blood and HF-brain (ANOVA, Bonferroni adjusted p<0.0028 and 0.0032, respectively) and selected examples in graphs below; +LPS, LPS-exposed progeny; up and downward-pointing arrowheads indicate direction of FA change across AA/SBO groups; f, females; m, males; *,**,***;****,*****, p<0.05, 0.01, 0.001, 10^−4^ and 10^−5^, respectively (Scheffé’s *post hoc*).

**Fig 6:**
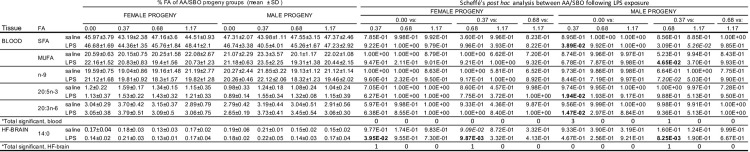
Scheffé’s post hoc analysis of offspring OFT behaviors that differed across saline-exposed and LPS-exposed AA/SBO groups. *Fatty acids (FA) that showed significance following ANOVA (Bonferroni adjusted p<0.0028 and 0.0031, respectively for individual FA, p<0.0167 for FA grouped by saturation) and Scheffé’s post hoc analysis (p<0.05, in bold); mean, n = 6/experimental group.

ANOVA of FA profiles of blood and HF-brain of female or male offspring showed no significant differences between saline-exposed and LPS-exposed AA/SBO experimental groups (**S2 Table and Fig 5)**. In contrast, five FA (SFA, MUFA, n-9, 20:3n-6 and 20:5n-3) and one FA (14:0) in blood and HF-brain, respectively, differed significantly across founder AA/SBO groups (**S2 Table)**. Blood FA were significant only in males, while 14:0 was significant in both sexes in HF-brain **(Fig 5**). *Post hoc* analysis showed that significance in blood and brain was restricted to LPS-exposed progeny (**Fig 6**). In blood, male-specific differences were between AA/SBO 0.00 and 0.37 (SFA, 20:3n-6 and 20:3n-3) or between AA/SBO 0.37 and 0.68 (MUFA). In the former comparison, 0.37 AA/SBO progeny showed a reduction in SFA compared to 0.00 progeny, while 20:3n-6 and 20:3n-3 showed the opposite effect; in the latter, MUFA were increased in AA/SBO 0.37 relative to 0.68 progeny (**Figs 3** and **6**). In the brain, 14:0 differed significantly between AA/SBO 0.37 and AA/SBO 0.68 for both sexes and both showed decreased levels in 0.68 AA/SBO progeny (**Figs 3** and **6**). In addition, 14:0 in female progeny also differed significantly between AA/SBO 0.00 and 0.37, with an increase in the latter.

**Fig 7:**
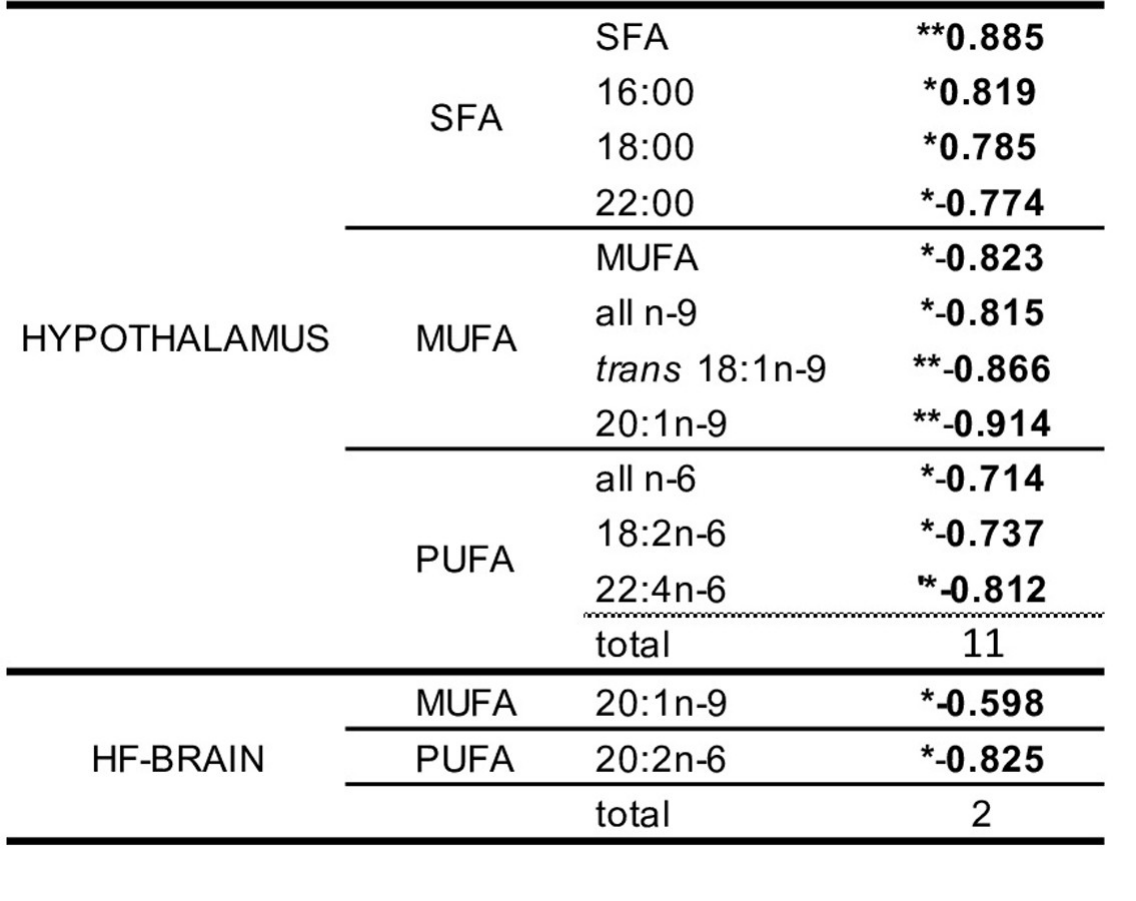
Correlations between blood, HF-brain and hypothalamus FA and founder AA/SBO. In bold, significant correlations (Pearson’s, *p<0.05, **p<0.01, ***p<0.001) between founder AA/SBO and FA content across all offspring (i.e. male and female offspring as one experimental group).

Akin to blood and HF-brain, paired *t*-test analysis of hypothalamic FA content between saline-exposed and LPS-exposed offspring revealed no significant differences. Notably, most FA (11/13) showed significant correlations with founder AA/SBO (**Fig 7**). A similar analysis performed in blood and HF-free brain revealed none and two, respectively. In both HF-brain and hypothalamus 20:1n-9 correlated negatively with founder AA/SBO.

Taken together, the data show that alterations in AA/SBO were associated with tissue-specific and sex-specific FA profiles. With respect to the latter, associations with founder AA/SBO exposure were evident in blood, but not in HF-brain.

### Associations between founder AA/SBO dependent offspring behavior and gene expression in the hypothalamus

Finally, we asked whether founder AA/SBO dependent OFT behavior was associated with changes in expression of genes related to inflammation and FA synthesis. To this end, we focused on the hypothalamus given its importance in anxiety disorders [25].

With respect to inflammation, we analyzed the expression profiles of two proinflammatory genes, *Tnfa* and *Il1b*, that have been previously shown to be upregulated in the hypothalamus following a 2-hour LPS exposure [22], in addition to *Cox1* and *Cox2*, that play a central role in inflammatory processes by converting AA into bioactive prostanoids [26]. Furthermore, based on the observation that founder AA/SBO correlated positively and negatively with SFA and MUFA, we analyzed the expression profile of *Scd1*, that catalyzes the endogenous synthesis of MUFA (16:1*n*-7 and 18:1*n*-9) from SFA (16:0 and 18:0), in addition to *Elovl6*, the main elongase that converts 16:0 to 18:0 [27, 28]. RT-PCR was performed on three individual hypothalami from male and female AA/SBO offspring groups and products were normalized to *M*. *musculus* ribosomal *36b4* expression.

**Fig 8:**
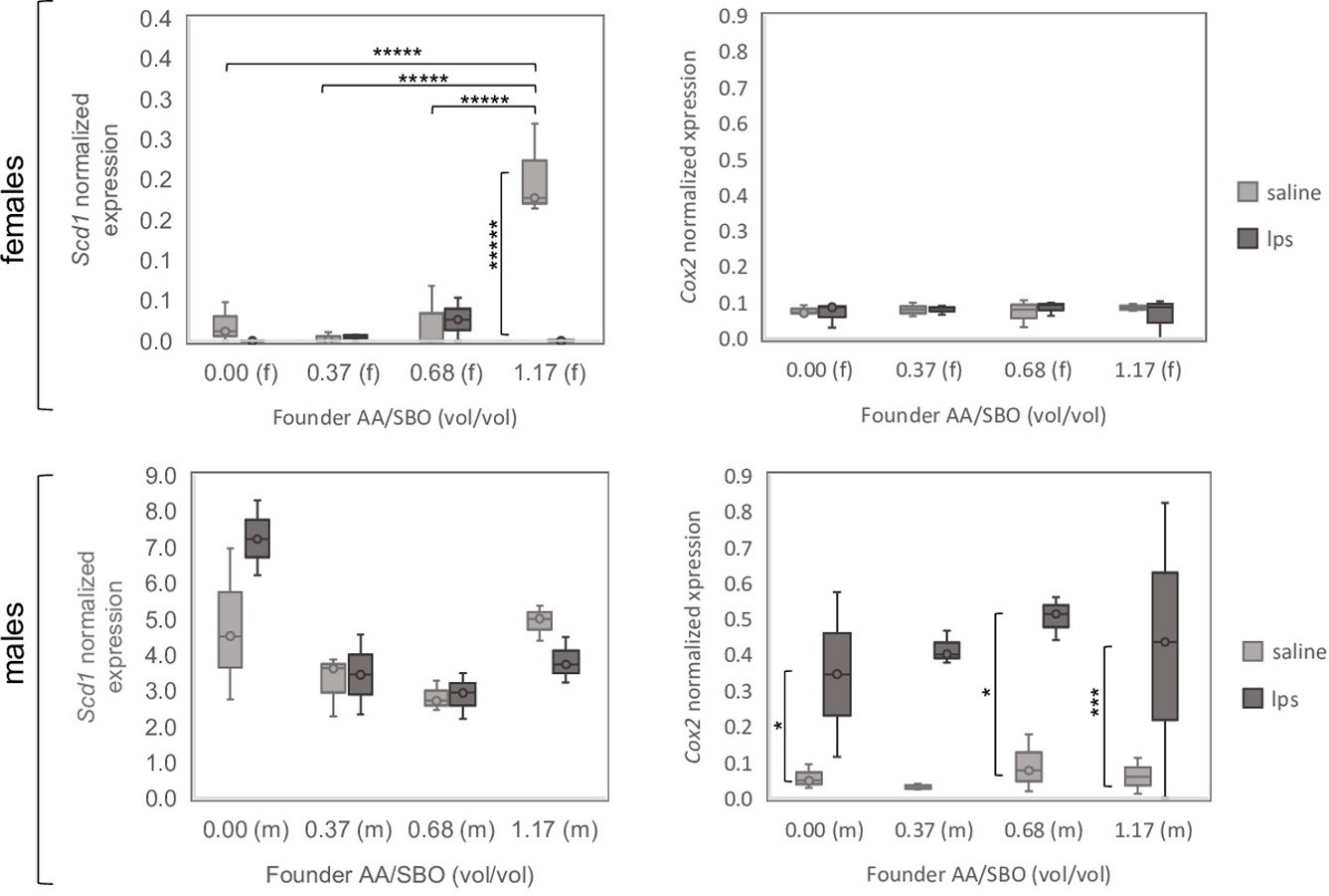
RT-PCR analysis of selected genes related to inflammation and fatty acid synthesis. Expression values were normalized to *36b4*; *,***, *****, *****, p<0.1, 0.05, 0.01, 10^−4^ and 10^−5^, respectively (Scheffé’s *post hoc*); n = 3/founder AA/SBO group.

In males, *Cox2* showed significant differences between saline-exposed and LPS-exposed groups in all AA/SBO groups, except the AA/SBO 0.37 group that showed borderline significance (**Fig 8, right panels; S2A Fig**). *Tnfa* and *Il1b* showed similar expression profiles to *Cox2*, albeit not significant (**S3 Fig**). *Elovl6* did not display any profile similarity nor significance. *Scd1* showed significant differences in hypothalamic expression across founder AA/SBO groups, specifically in females as follows: 1) reduced expression in AA/SBO 1.17 LPS group compared to its corresponding saline group (p<9.41E-9); 2) an increase in expression in AA/SBO 1.17 compared to 0.00, 0.37 and 0.68 saline groups (p = 6.28x10^-7^, 1.66x10^-7^ and 8.06x10^-7^, respectively). By contrast, no significant difference was observed across AA/SBO in males.

Finally, we asked if *Scd1* expression was related to the changes in FA profiles observed following AA/SBO treatment. Since FA profiles were characterized from hypothalami pooled from three male and three female individuals, RT-PCR was repeated on pooled samples in order to perform the correlative analysis (**S2B Fig**). However, we found no correlations between FA accumulation and gene expression profiles.

## Discussion

The main outcome of our study is a series of intergenerational associations between paternal supplementation with AA and the unsupplemented progeny’s response to LPS exposure. The paternally supplemented AA dose significantly interacted with LPS in affecting the progeny’s OFT behavior. LPS *per se* elicited progeny OFT behaviors consistent with increased anxiety—longer time spent in corners and less overall mobility. Paternal AA dose affected that response: a prominent pattern was the significant divergence of OFT outcome between LPS-exposed and saline-exposed groups in females, but their realignment at high AA/SBO in males. The data suggest that high paternal AA/SBO primes the male progeny acting as surrogate of LPS, essentially mimicking the latter in saline-exposed males.

The observed marked sexual dimorphism is consistent with the established evidence that males are comparatively more responsive to inflammatory stimuli and refractive to anti-inflammatory corrective treatments [29]. For example, LPS induces a markedly stronger expression of inflammatory cytokines in cortical primary astrocytes isolated from male compared to female mice, or female mice previously exposed to androgen [30]. Notably, AA supplementation in mice with diet-induced obesity exerts sex-specific effects on obesity-induced metabolic defects. AA provokes a shift towards a pro-inflammatory intestinal microbiota in males, but, strikingly, improves the metabolic profile and induces an anti-inflammatory microbiome in females [31]. Furthermore, the latter study showed increased levels of circulating LPS in obese mice exposed to AA, as a result of impaired intestinal barrier.

Non-linear associations were prominent in our findings. Similar non-linear intergenerational effects have been reported in different disease models and in humans, although the underlying mechanisms are poorly understood. As for metabolic disease, our observation that mid-range paternal AA doses tended to exert the strongest effects on OFT behavior, echoes the inverted U-shaped association between the extent of paternal dietary restriction and the level of fat content of progeny and similar intergenerational effects in *C*. *elegans* [32]. Additional examples of U-shaped responses include metabolic and psychiatric offspring phenotypes associated with maternal exposure-determined newborn birth weight, paternal diet or age in *D*. *melanogaster* and humans [33, 34].

Progeny FA were generally insensitive to LPS treatment, but selected ones associated with paternal AA/SBO, particularly blood FA in LPS-exposed males. The former result reinforces the idea that the significant differences observed in our study do not arise from random variation between experiments. Yet, after dissecting the association between FA and paternal AA dose in LPS and saline controls separately, we concluded that paternal AA primed the male progeny for a relatively fast response to LPS in terms of FA metabolism in peripheral blood. Similar conclusions can be reached for HF-brain, but with a less clear sex specificity. We detected associations between paternal AA supplementation and progeny brain FA that largely reflect published evidence. Particularly, the microbiome is emerging as a functional link between the diet and brain FA, pointing to speculative candidate mechanisms underlying our findings. One notable case is the negative correlation of brain 20:1n-9 with paternal AA in the hypothalamus and HF-brain of female progeny in our model. Brain FA were dramatically altered in a mouse model of old-to-young microbiome transfer; particularly, brain AA was negatively associated with 20:1n-9, a highly represented FA in the brain [35]. Likely reflecting exposure-specific effects, the female progeny-specific positive association of 18:1n-9 with paternal AA detected in our model, is not consistent with the outcome of that same study. Other reports suggest that reduced C20:1n-9 levels in serum are associated with markers of inflammation and metabolically unhealthy obesity, providing further context for our data: blood cell membrane 20:1n-9 and MUFA were negatively associated with a range of cytokines at baseline in a large human association study within the PREDIMED cohort; 14:0 showed negative associations both at baseline and after a 1-year follow-up [36]. Interestingly, 14:0 exerts marked behavioral effects in experimental models: it showed anxiolytic effects comparable to the drug diazepam in a rat model [37]. To our knowledge, it is not clear whether these functions of 14:0 are related to its unique characteristics among FA: a marked underrepresentation in cellular membrane and a prominent role in protein posttranslational modification [38]. Furthermore, 14:0 or SFA contribute to learning and memory formation [39]. In blood, AA exposure negatively affected 20:5n-3 (EPA). Likewise, a reduction in EPA content with increasing concentrations of AA in blood has been observed in plasma and milk following human dietary interventions with AA [14].

Notably, hypothalamic expression of *Scd1* was affected by paternal AA exposure in female saline exposed offspring, but not the inflammatory markers *Il1b*, *Tnfa*. The latter suggests that the effect of paternal AA on the progeny’s anxiety-related behavior is dissociated from its effects on inflammatory markers. The pattern of *Scd1* expression is particularly interesting. High AA/BSO primes females for high *Scd1*, but LPS blunts that response. As high SCD1 is considered proinflammatory and promoting cognitive impairment, the data suggest that high AA/SBO primes for SCD1-mediated inflammation, but on a distinct pathway than LPS [40]. By contrast hypothalamic *Cox2*—a generator of proinflammatory mediators from AA—was increased by LPS in males, but not in females, and largely independent of paternal AA supplementation. The marked response by *Cox2* to LPS is in line with previous observations [41]. A further dissociation is between the AA/SBO effects on pro-inflammatory cytokine gene expression and the generation of AA-derived inflammatory mediators (*Cox2* expression)—in saline-exposed and in LPS-exposed group, respectively. Keeping in mind those *caveats*, our data indicate a net shift towards a pro-inflammatory profile and reduced tolerance to LPS in the progeny with increased paternal AA/SBO.

Our data mirror the well documented associations between parental diet exposure and offspring anxiety levels in rodents. Most studies have focused on the effects of maternal high-fat diet (HFD) during mating, pregnancy, and lactation and have demonstrated adverse effects on anxiety levels not only in F1 offspring, but also in F2 and possibly F3 generations [42, 43]. Although corresponding data pertaining to paternal exposure is limited [20], the association of paternal stress, trauma, HFD and environmental toxins with aberrant offspring behaviors, such as anxiety, risk taking, memory deficits and social behavior has been documented [44–49]. Our data are consistent with the general observation that paternal exposure can alter the immune response, although with complex outcomes: on the one hand, offspring of male mice exposed to sublethal amounts of *C*. *albicans* inherited an augmented response to infections compared with progeny of untreated males [50]. On the other hand, a further adult exposure to LPS induced opposite effects on pro-inflammatory cytokines in microglia from different locations of the brain in a model of pro-inflammatory exposure *in utero* [51].

Our experimental model was based on determining the effects of three paternal doses of AA on offspring OFT behavior and tissue-specific FA. To maintain total number of total calories consumed, AA supplements were administered together with SBO. A limitation of this approach is that causality cannot be unequivocally assigned to founder AA. However, some observations support an active role of founder AA in offspring phenotypes. Firstly, AA affects EPA content negatively in blood—both in our model and in human dietary interventions with AA [14]. Secondly, in our previous experiment that compared cumulative effects of an AA + SBO supplement—or SBO alone—across three offspring generations, only mice exposed to the former showed correlations with FA content [17]. Notably, in that study EPA liver content correlated with paternal and maternal AA exposure, albeit positively. Interestingly, akin to our observation that AA may be a more potent modulator of FA content compared to FA present in SBO, diets rich in n-3 FA (e.g. EPA and DHA) result in more significant alterations in brain fat content compared to diets rich in SFA or PUFA [52].

Due to the significant gaps in the knowledge of intergenerational paternal inheritance, the mechanisms underlying the responses observed in the present study can only be speculated. Supplemented AA may modulate brain FA and inflammation markers by increasing the gut permeability to LPS as a result of remodeling the microbiome in favor of Gram-negative bacteria [31]. A complementary mechanism may involve increased conversion of AA proinflammatory mediators, as AA metabolism is highly active in microglia [52]. How the above-mentioned findings, however mechanistically pertinent, can be applied to our model of paternal inheritance can only be speculated. AA supplementation may generate sperm loaded with signals—transcripts, transcriptional regulators—that prime the embryo to initiate and sustain the production of inflammation-related FA and cytokines. These events may be followed by a bias in favor of Gram-negative bacteria in the postnatal microbiome. Rapid responses of the sperm transcriptome to a high-sugar diet have been described [53]. Interestingly, a prime role for FA, particularly the ones acting as ligands for peroxisome proliferator activated receptors, has been shown in a model paternal inheritance of traumatic stress [54]. Other components of the ejaculate may participate in these phenomena, by modulating inflammation in the uterine wall [55]. The molecular nature of the sustained—i.e., resistant to dilution in the highly proliferating embryo and fetus—anti- or proinflammatory program inherited following paternal exposure, is a poorly understood key information. Based on current understanding of transcription regulation, it is conceivable that paternal AA supplementation imposes highly stable and mitotically inherited specific chromatin states in critical loci involved in FA metabolism and cytokine synthesis in the progeny.

## Supporting information

S1 Table OFT behaviors successfully determined by the ANY-maze software. (PDF)

S2 Table Offspring OFT behaviors and FA that differed across and within LPS-exposed and saline-exposed AA/SBO groups. (PDF)

S3 Table Correlations between female and male offspring OFT behaviors across saline-exposed and LPS-exposed AA/SBO groups. (PDF)

S1 Fig Effects of founder AA/SBO on founder and offspring body weight. Light and dark grey boxes, body weight of male founders (n = 3) and body weight of their offspring (n = 12, 6 females and 6 males per founder male), respectively. ANOVA and Scheffé’s post hoc.
(PDF)

S2 Fig RT-PCR analysis of selected genes related to inflammation and fatty acid synthesis. A) Three individual hypothalami from AA/SBO male and female experimental groups; original gels are shown in S1 and S2_raw_images in S1 Raw images. B) Pooled sample of RNA from 3 male and three female hypothalami i.e. samples shown in A; original gels are shown in S3_raw_image in S1 Raw images.
(PDF)

S3 Fig RT-PCR analysis of selected genes related to inflammation and fatty acid synthesis. Expression values of each gene were normalized to 36b4; f and m, female and male, respectively; asterisks indicate significance in post hoc analysis; *,***, *****, *****, p<0.1, 0.05, 0.01, 0.0001 and 0.00001, respectively; (n = 3/founder AA/SBO group).
(PDF)

S1 Raw images (PDF)
