# Correction: Effects of paternal arachidonic acid supplementation on offspring behavior and hypothalamus inflammation markers in the mouse

**DOI:** 10.1371/journal.pone.0307828

**Published:** 2024-07-22

**Authors:** Atenea Vázquez-Sánchez, Dalia Rodríguez-Ríos, Dannia Colín-Castelán, Jorge Molina-Torres, Enrique Ramírez-Chávez, Gloria del Carmen Romo-Morales, Silvio Zaina, Gertrud Lund

There are errors in Figs 2 to 6. In Fig 2, the ANOVA female, Founder AA/SBO should have been 0 not 1. Consequently, the Figs 3, 4, and 6 are uploaded incorrectly.

In Fig 5, the Y-axis of blood should be "% SFA/total FA" not "% SFA and MUFA/total FA". Then, the FOUNDER AA/SBO in males in the Venn diagram should be 5 not 4. Meanwhile, the FOUNDER AA/SBO in female and males in Venn diagram of HF-brain is 1 and 1, respectively.

**Fig 2:**
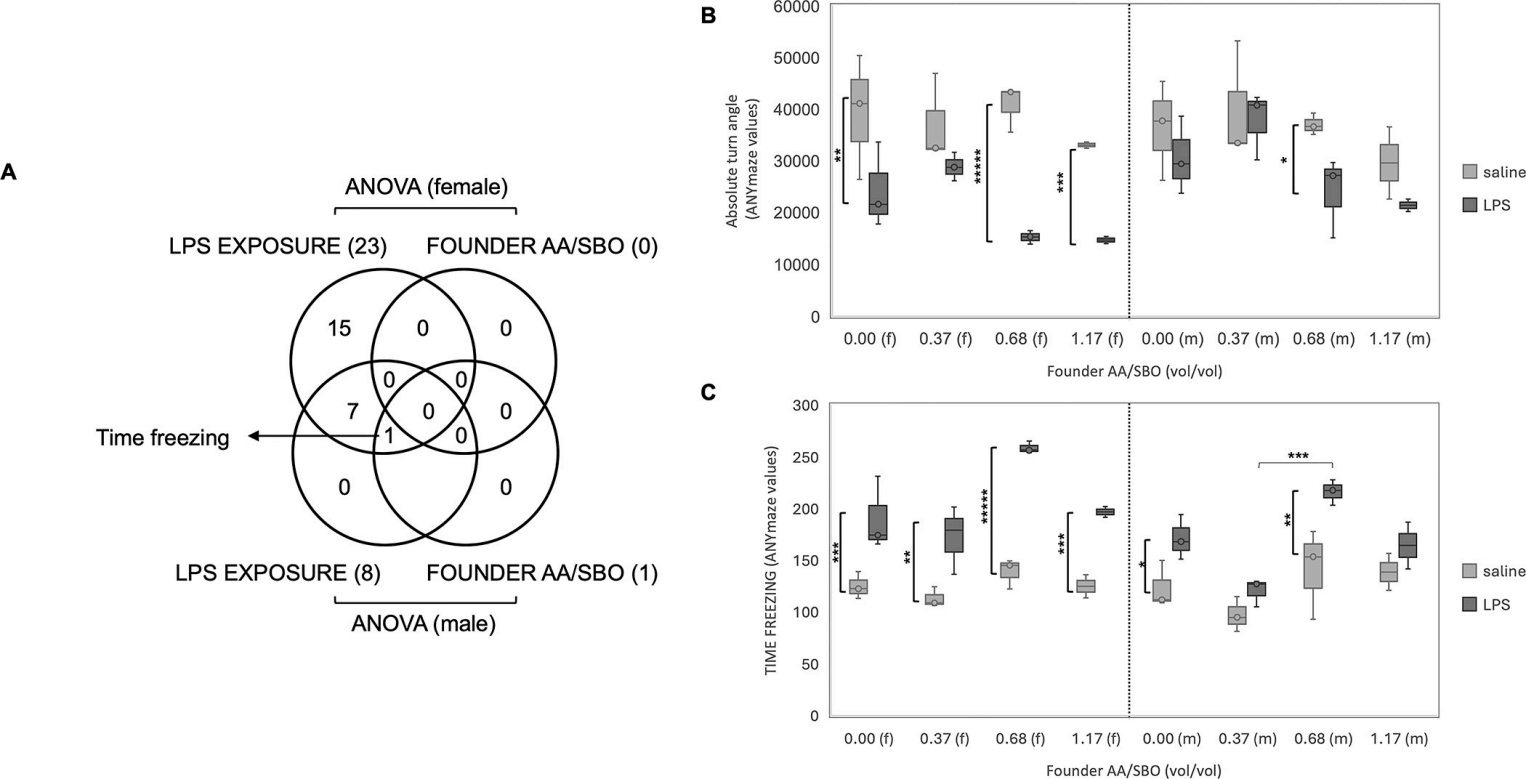
Female and male OFT behaviors associated with LPS exposure and/or founder AA/SBO. A) Venn diagrams of significant OFT behaviors in female and male progeny (ANOVA, Bonferroni adjusted p<0.00167); B) Representative OFT behavior that responded more significantly to LPS exposure in female compared to male offspring; C) OFT behavior that differed across AA/SBO 1.17 in LPS-exposed males; f, females, m, males; *,**,***;****,*****, p<0.05, 0.01, 0.001, 10^−4^ and 10^−5^, respectively (Scheffé’s *post hoc* and t-test; solid and dotted brackets, respectively). https://doi.org/10.1371/journal.pone.0300141.g002.

**Fig 3:**
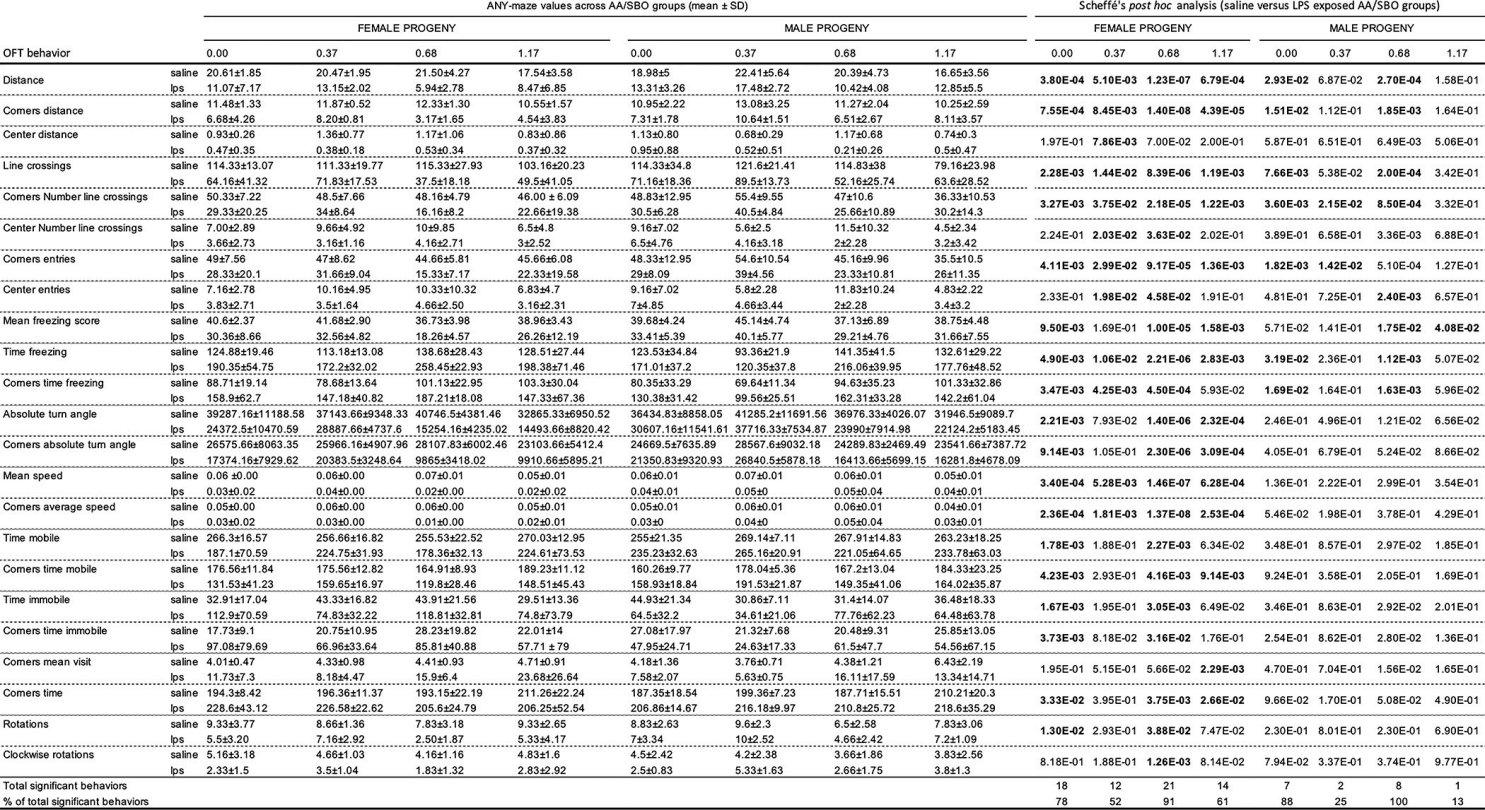
Scheffé’s post hoc analysis of progeny OFT behaviors that differed across saline-exposed and LPS-exposed AA/SBO groups. *OFT behaviors that showed significance following ANOVA (Bonferroni adjusted p<0.00167) and Scheffé’s post hoc analysis (p<0.05, in bold); mean, n = 6/experimental group. https://doi.org/10.1371/journal.pone.0300141.g003.

**Fig 4:**
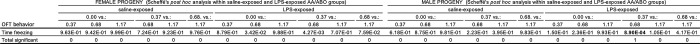
Scheffé’s post hoc analysis of progeny OFT behaviors affected by founder AA/SBO treatment. *OFT behaviour that showed significance following ANOVA (Bonferroni adjusted p<0.00167) and Scheffé’s post hoc analysis (p<0.05, in bold). https://doi.org/10.1371/journal.pone.0300141.g004.

**Fig 5:**
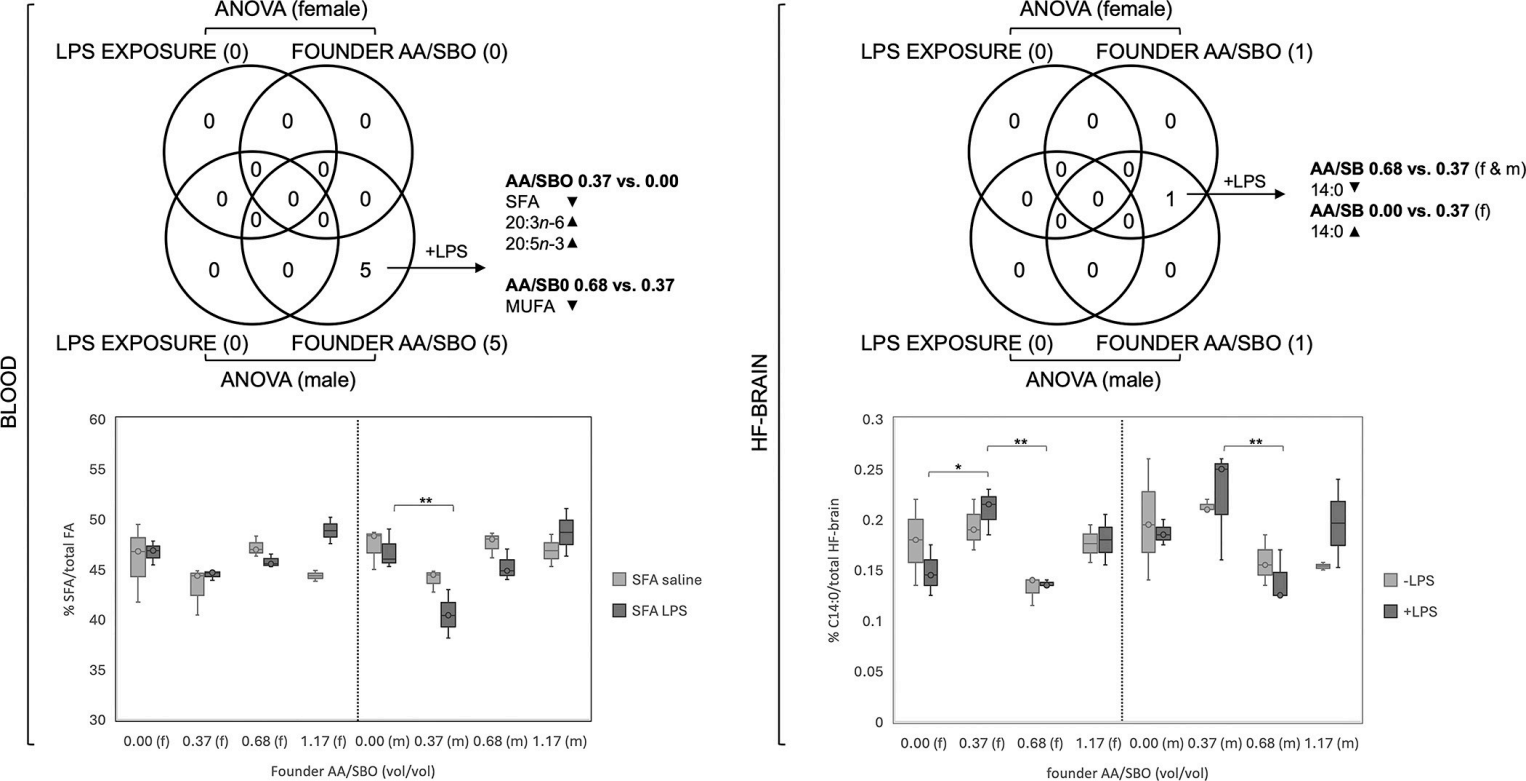
Female and male blood and HF-brain FA that were associated with LPS exposure and/or founder AA/SBO. Venn diagrams of significant FA in blood and HF-brain (ANOVA, Bonferroni adjusted p<0.0028 and 0.0032, respectively) and selected examples in graphs below; +LPS, LPS-exposed progeny; up and downward-pointing arrowheads indicate direction of FA change across AA/SBO groups; f, females; m, males; *,**,***;****,*****, p<0.05, 0.01, 0.001, 10^−4^ and 10^−5^, respectively (Scheffé’s *post hoc*). https://doi.org/10.1371/journal.pone.0300141.g005.

**Fig 6:**
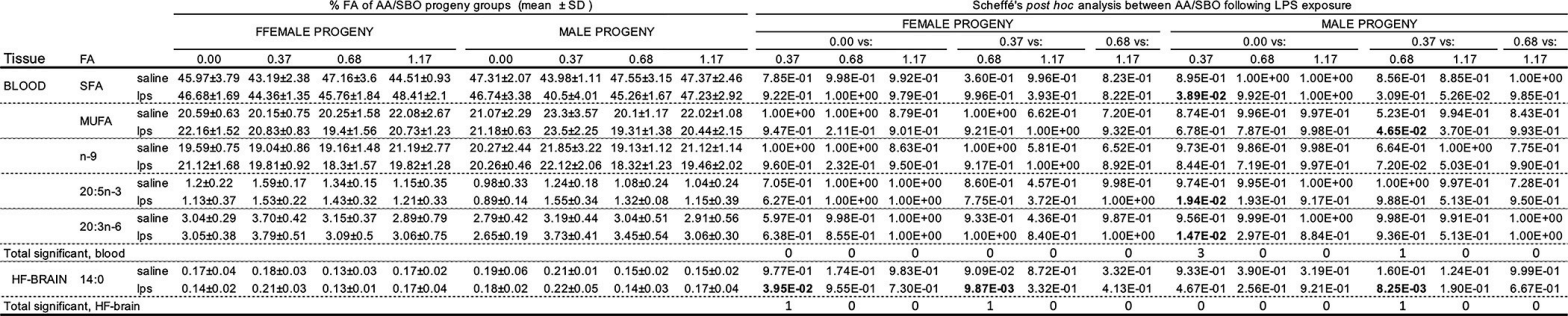
Scheffé’s post hoc analysis of offspring OFT behaviors that differed across saline-exposed and LPS-exposed AA/SBO groups. *Fatty acids (FA) that showed significance following ANOVA (Bonferroni adjusted p<0.0028 and 0.0031, respectively for individual FA, p<0.0167 for FA grouped by saturation) and Scheffé’s post hoc analysis (p<0.05, in bold); mean, n = 6/experimental group. https://doi.org/10.1371/journal.pone.0300141.g006.

Moreover, the S2 and S3 Tables are uploaded incorrectly. The authors have provided the correct version of Figs 2 to 6 and S2 and S3 Tables here.
