# Supplementary material for: Effects of paternal arachidonic acid supplementation on offspring behavior and hypothalamus inflammation markers in the mouse
Source: PLoS One. 2024 Mar 21;19(3):e0300141. doi: 10.1371/journal.pone.0300141 (PMC10956830; doi:10.1371/journal.pone.0300141)
Supplement: S1 Table — (PDF) [file pone.0300141.s001.pdf]

S1 Table - OFT behaviors successfully determined by the ANYmaze software.

Distance
Corners distance
Center distance
Line crossings
Corners Number line crossings
Center Number line crossings
Corners entries
Center entries
Mean freezing score
Time freezing
Corners time freezing
Center time freezing
Absolute turn angle
Corners absolute turn angle
Center absolute turn angle
Mean speed
Corners average speed
Time mobile
Corners time mobile
Center time mobile
Time immobile
Corners time immobile
Center time immobile
Corners mean visit
Corners time
Center time
Rotations
Anticlockwise rotations
Clockwise rotations
Num centre positions
