# Supplementary material for: Effects of paternal arachidonic acid supplementation on offspring behavior and hypothalamus inflammation markers in the mouse
Source: PLoS One. 2024 Mar 21;19(3):e0300141. doi: 10.1371/journal.pone.0300141 (PMC10956830; doi:10.1371/journal.pone.0300141)
Supplement: S2 Table — (PDF) [file pone.0300141.s002.pdf]

S2 Table - *Post hoc* analysis of offspring OFT behaviours that differed across saline and LPS-exposed AA/SBO offspring groups.

	ANOVA (saline-exposed compared to LPS-exposed mice)														
		Scheffe's *post hoc*, all (AA/SBO)					Scheffe's *post hoc*, female (AA/SBO)					Scheffe's *post hoc*, males (AA/SBO)			
	all	0.00 vs 0.00	0.37 vs 0.37	0.68 vs 0.68	1.17 vs 1.17	female	0.00 vs 0.00	0.37 vs 0.37	0.68 vs 0.68	1.17 vs 1.17	male	0.00 vs 0.00	0.37 vs 0.37	0.68 vs 0.68	1.17 vs 1.17
Distance	**4.65E-14**	***5.64E-05***	***1.48E-03***	***2.76E-10***	***5.15E-04***	**2.79E-10**	***3.80E-04***	***5.10E-03***	***1.23E-07***	***6.79E-04***	**4.00E-05**	***2.93E-02***	6.87E-02	***2.70E-04***	1.58E-01
Corners distance	**1.23E-13**	***8.56E-05***	***5.29E-03***	***1.15E-09***	***1.13E-04***	**5.37E-11**	***7.55E-04***	***8.45E-03***	***1.40E-08***	***4.39E-05***	**1.10E-04**	***1.51E-02***	1.12E-01	***1.85E-03***	1.64E-01
Center distance	**6.00E-05**	1.87E-01	***1.75E-02***	***1.23E-03***	1.58E-01	**8.15E-04**	1.97E-01	***7.86E-03***	7.00E-02	2.00E-01	2.72E-02	5.87E-01	6.51E-01	***6.49E-03***	5.06E-01
Line crossings	**4.59E-13**	***4.03E-05***	***1.92E-03***	***4.20E-09***	***1.96E-03***	**1.26E-08**	***2.28E-03***	***1.44E-02***	***8.39E-06***	***1.19E-03***	**3.00E-05**	***7.66E-03***	5.38E-02	***2.00E-04***	3.42E-01
Corners Number line crossings	**1.71E-12**	***3.45E-05***	***2.45E-03***	***5.86E-08***	***1.54E-03***	**4.76E-08**	***3.27E-03***	***3.75E-02***	***2.18E-05***	***1.22E-03***	**2.00E-05**	***3.60E-03***	***2.15E-02***	***8.50E-04***	3.32E-01
Center Number line crossings	**5.41E-05**	1.37E-01	***4.54E-02***	***2.34E-04***	2.42E-01	**1.02E-03**	2.24E-01	***2.03E-02***	***3.63E-02***	2.02E-01	2.06E-02	3.89E-01	6.58E-01	***3.36E-03***	6.88E-01
Corners entries	**5.81E-13**	***2.31E-05***	***1.56E-03***	***1.40E-07***	***4.87E-04***	**1.00E-07**	***4.11E-03***	***2.99E-02***	***9.17E-05***	***1.36E-03***	**3.86E-06**	***1.82E-03***	***1.42E-02***	***5.10E-04***	1.27E-01
Center entries	**7.00E-05**	1.78E-01	5.07E-02	***2.35E-04***	2.19E-01	**1.15E-03**	2.33E-01	***1.98E-02***	***4.58E-02***	1.91E-01	2.29E-02	4.81E-01	7.25E-01	***2.40E-03***	6.57E-01
Mean freezing score	**1.13E-09**	***2.18E-03***	6.92E-02	***2.29E-06***	***2.68E-04***	**2.69E-07**	***9.50E-03***	1.69E-01	***1.00E-05***	***1.58E-03***	**4.60E-04**	5.71E-02	1.41E-01	***1.75E-02***	***4.08E-02***
Time freezing	**7.40E-12**	***5.43E-04***	***1.05E-02***	***1.79E-08***	***4.69E-04***	**1.39E-08**	***4.90E-03***	***1.06E-02***	***2.21E-06***	***2.83E-03***	**1.20E-04**	***3.19E-02***	2.36E-01	***1.12E-03***	5.07E-02
Corners time freezing	**9.88E-11**	***1.60E-04***	***2.37E-03***	***2.36E-06***	***7.50E-03***	**6.49E-07**	***3.47E-03***	***4.25E-03***	***4.50E-04***	5.93E-02	**7.00E-05**	***1.69E-02***	1.64E-01	***1.63E-03***	5.96E-02
Absolute turn angle	**5.26E-10**	***3.55E-03***	1.10E-01	***2.72E-07***	***9.72E-05***	**7.52E-09**	***2.21E-03***	7.93E-02	***1.40E-06***	***2.32E-04***	4.90E-03	2.46E-01	4.96E-01	***1.21E-02***	6.56E-02
Corners absolute turn angle	**2.63E-08**	***2.05E-02***	1.96E-01	***3.77E-06***	***2.00E-04***	**3.18E-08**	***9.14E-03***	1.05E-01	***2.30E-06***	***3.09E-04***	2.44E-02	4.05E-01	6.79E-01	5.24E-02	8.66E-02
Mean speed	**1.37E-08**	***1.49E-03***	***1.40E-02***	***7.72E-05***	***6.88E-03***	**2.80E-10**	***3.40E-04***	***5.28E-03***	***1.46E-07***	***6.28E-04***	2.93E-02	1.36E-01	2.22E-01	2.99E-01	3.54E-01
Corners average speed	**6.97E-09**	***5.75E-04***	***9.43E-03***	***9.18E-05***	***9.09E-03***	**2.94E-11**	***2.36E-04***	***1.81E-03***	***1.37E-08***	***2.53E-04***	2.14E-02	5.46E-02	1.98E-01	3.78E-01	4.29E-01
Time mobile	**2.12E-06**	***3.10E-03***	2.99E-01	***2.53E-04***	***2.54E-02***	**1.65E-05**	***1.78E-03***	1.88E-01	***2.27E-03***	6.34E-02	2.66E-02	3.48E-01	8.57E-01	***2.97E-02***	1.85E-01
Corners time mobile	**1.10E-04**	***3.16E-02***	9.20E-01	***3.87E-03***	***5.03E-03***	**1.60E-05**	***4.23E-03***	2.93E-01	***4.16E-03***	***9.14E-03***	3.89E-01	9.24E-01	3.58E-01	2.05E-01	1.69E-01
Time immobile	**2.51E-06**	***2.82E-03***	3.06E-01	***3.21E-04***	***2.76E-02***	**1.99E-05**	***1.67E-03***	1.95E-01	***3.05E-03***	6.49E-02	2.78E-02	3.46E-01	8.63E-01	***2.92E-02***	2.01E-01
Corners time immobile	**5.77E-06**	***2.40E-03***	1.34E-01	***2.80E-03***	5.17E-02	**1.39E-04**	***3.73E-03***	8.18E-02	***3.16E-02***	1.76E-01	1.68E-02	2.54E-01	8.62E-01	***2.80E-02***	1.36E-01
Corners mean visit	**2.91E-05**	1.34E-01	4.53E-01	***2.18E-03***	***5.85E-04***	**9.25E-04**	1.95E-01	5.15E-01	5.66E-02	***2.29E-03***	1.93E-02	4.70E-01	7.04E-01	***1.56E-02***	1.65E-01
Corners time	**9.32E-04**	***8.86E-03***	***2.37E-02***	8.07E-02	9.13E-01	**1.45E-04**	***3.33E-02***	3.95E-01	***3.75E-03***	***2.66E-02***	1.60E-01	9.66E-02	1.70E-01	5.08E-02	4.90E-01
Rotations	**1.56E-04**	***1.65E-02***	6.70E-01	***2.65E-03***	***4.58E-02***	**4.84E-04**	***1.30E-02***	2.93E-01	***3.88E-02***	7.47E-02	4.63E-03	2.30E-01	8.01E-01	2.30E-01	6.90E-01
Clockwise rotations	**1.26E-03**	***2.29E-03***	9.62E-01	***3.31E-02***	1.81E-01	**1.57E-03**	8.18E-01	1.88E-01	***1.26E-03***	8.14E-02	2.18E-01	7.94E-02	3.37E-01	3.74E-01	9.77E-01
	23	19	12	22	17	23	18	12	21	14	8	7	2	16	0

In bold and "blod+italics"; p<0.00167 and p<0.05 ((ANOVA, Bonferroni adjusted p and Scheffe's post hoc, respectively); all, pooled male and female offspring.
