# Supplementary material for: Effects of paternal arachidonic acid supplementation on offspring behavior and hypothalamus inflammation markers in the mouse
Source: PLoS One. 2024 Mar 21;19(3):e0300141. doi: 10.1371/journal.pone.0300141 (PMC10956830; doi:10.1371/journal.pone.0300141)
Supplement: S3 Table — (PDF) [file pone.0300141.s003.pdf]

S3 Table - *Post hoc* analysis of offspring OFT behaviours affected by founder AA/SBO exposure.

		all offspring (Scheffe's *post hoc* analysis of founder AA/SBO)											
		saline-exposed						LPS-exposed					
		0.00 vs.:			0.37 vs.:		0.68 vs.:	0.00 vs.:			0.37 vs.:		0.68 vs.:
	all	0.37	0.68	1.17	0.68	1.17	1.17	0.37	0.68	1.17	0.68	1.17	1.17
Time freezing	**6.57E-06**	6.73E-01	8.00E-01	9.83E-01	1.83E-01	4.47E-01	9.48E-01	1.97E-01	***6.99E-03***	9.66E-01	***2.82E-06***	7.81E-02	***3.51E-02***
Mean freezing score	**3.00E-05**	7.04E-01	6.69E-01	9.69E-01	1.29E-01	4.26E-01	9.04E-01	3.97E-01	***2.21E-02***	6.91E-01	***8.42E-05***	***4.39E-02***	3.14E-01
Absolute turn angle	**4.70E-04**	9.91E-01	9.94E-01	4.83E-01	1.00E+00	3.28E-01	3.31E-01	4.26E-01	1.69E-01	7.22E-02	***2.35E-03***	***6.69E-04***	9.74E-01
Anticlockwise rotations	**1.43E-03**	9.71E-01	6.42E-01	1.00E+00	3.82E-01	9.71E-01	6.42E-01	9.79E-01	***1.84E-02***	7.12E-01	***5.40E-03***	4.70E-01	2.68E-01
Rotation	**8.85E-04**	1.00E+00	4.39E-01	9.80E-01	4.56E-01	9.80E-01	6.85E-01	2.64E-01	1.60E-01	1.00E+00	***7.11E-04***	2.58E-01	1.95E-01
Total significant	**5**	0	0	0	0	0	0	0	3	0	5	2	1

		female offspring (Scheffe's *post hoc* analysis of founder AA/SBO)											
		saline-exposed						LPS-exposed					
		0.00 vs.:			0.37 vs.:		0.68. vs.:	0.00 vs.:			0.37 vs.:		0.68 vs.:
	female	0.37	0.67	1.17	0.68	1.17	1.17	0.37	0.68	1.17	0.68	1.17	1.17
Time freezing	7.64E-03	9.63E-01	9.42E-01	9.99E-01	7.24E-01	9.23E-01	9.76E-01	8.79E-01	***3.42E-02***	9.88E-01	***4.27E-03***	7.07E-01	7.59E-02
Mean freezing score	2.88E-03	9.93E-01	7.64E-01	9.76E-01	5.98E-01	9.02E-01	9.43E-01	9.45E-01	***1.69E-02***	7.30E-01	***3.38E-03***	3.91E-01	1.91E-01
Absolute turn angle	2.79E-02	9.74E-01	9.92E-01	5.87E-01	8.93E-01	8.34E-01	4.12E-01	8.10E-01	2.83E-01	2.18E-01	***4.41E-02***	***3.01E-02***	9.99E-01
Anticlockwise rotations	2.05E-01	9.99E-01	9.82E-01	9.94E-01	9.94E-01	9.82E-01	9.23E-01	9.82E-01	2.46E-01	9.59E-01	1.19E-01	8.17E-01	5.17E-01
Rotation	1.36E-01	9.86E-01	8.64E-01	1.00E+00	9.73E-01	9.86E-01	8.64E-01	8.23E-01	4.09E-01	1.00E+00	8.24E-02	7.77E-01	4.60E-01
Total significant	0	0	0	0	0	0	0	0	2	0	3	1	0

		male offspring (Scheffe's *post hoc* analysis of founder AA/SBO)											
		saline-exposed						LPS-exposed					
		0.00 vs.:			0.37 vs.:		0.68. vs.:	0.00 vs.:			0.37 vs.:		0.68 vs.:
	male	0.37	0.68	1.17	0.68	1.17	1.17	0.37	0.68	1.17	0.68	1.17	1.17
Time freezing	**7.00E-04**	6.18E-01	8.75E-01	9.81E-01	2.23E-01	3.95E-01	9.83E-01	1.50E-01	2.36E-01	9.93E-01	***8.90E-04***	1.05E-01	4.17E-01
Mean freezing score	2.74E-03	1.13E-01	5.35E-01	9.78E-01	***3.56E-03***	5.16E-02	7.94E-01	4.61E-01	8.88E-01	9.94E-01	1.46E-01	3.20E-01	9.68E-01
Absolute turn angle	1.69E-02	8.32E-01	1.00E+00	8.45E-01	8.76E-01	3.70E-01	7.94E-01	5.66E-01	6.23E-01	4.56E-01	6.82E-02	***4.14E-02***	9.88E-01
Anticlockwise rotations		1.12E-01	6.97E-01	5.03E-01	5.99E-01	7.78E-01	9.89E-01	7.12E-02	5.99E-01	1.01E-01	6.73E-01	9.99E-01	7.61E-01
Rotation	4.63E-03	2.92E-01	8.53E-01	5.01E-01	7.42E-01	9.71E-01	9.31E-01	***1.12E-02***	4.71E-01	5.01E-01	3.81E-01	2.80E-01	9.99E-01
Total significant	1	0	0	0	1	0	0	1	0	0	1	1	0

In bold and "bold+italics"; p<0.00167 and p<0.05 (ANOVA, Bonferroni adjusted p and Scheffe's post hoc, respectively); all, pooled male and female offspring, light and dark grey cell, saline and LPS exposed offspring, respectively
