# Supplementary material for: Effects of paternal arachidonic acid supplementation on offspring behavior and hypothalamus inflammation markers in the mouse
Source: PLoS One. 2024 Mar 21;19(3):e0300141. doi: 10.1371/journal.pone.0300141 (PMC10956830; doi:10.1371/journal.pone.0300141)
Supplement: S1 Fig — Light and dark grey boxes, body weight of male founders (n = 3) and body weight of their offspring (n = 12, 6 females and 6 males per founder male), respectively. ANOVA and Scheffé’s post hoc. (PDF) [file pone.0300141.s004.pdf]

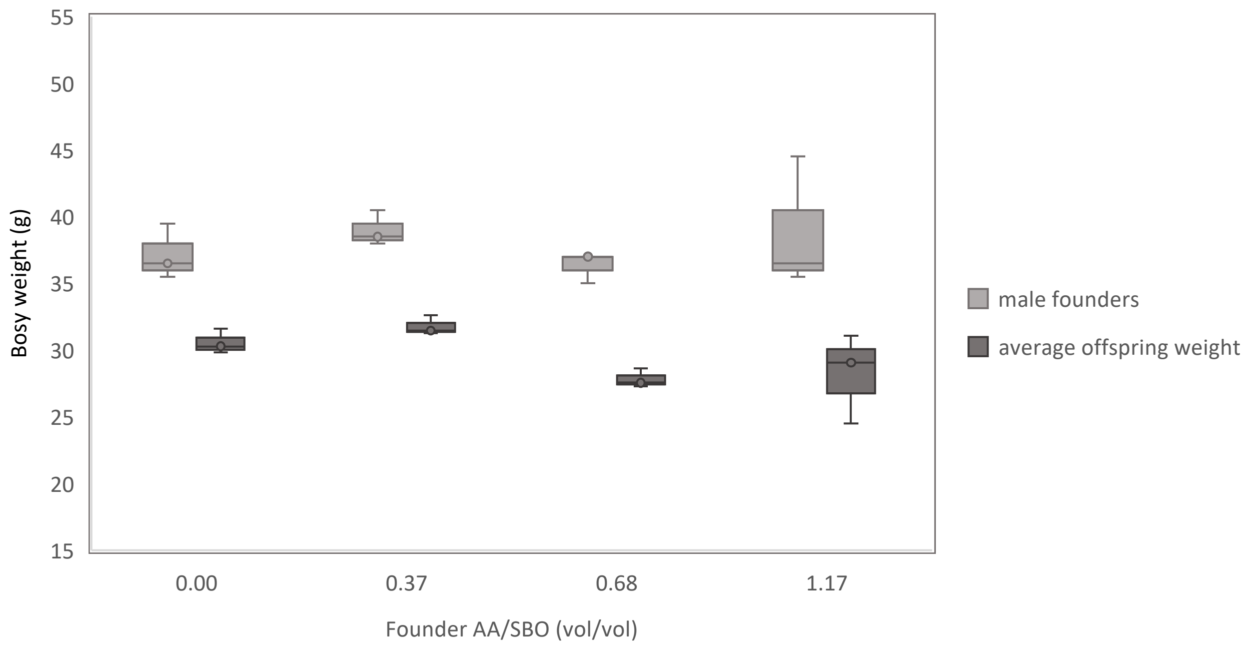

Supplemental Fig 1 - Effects of founder AA/SBO on founder and offspring body weight. Light and dark grey boxes, body weight of male founders (n=3) and body weight of their offspring (n=12, 6 females and 6 males per founder male), respectively. ANOVA and Scheffé's *post hoc*.
