# Supplementary material for: Effects of paternal arachidonic acid supplementation on offspring behavior and hypothalamus inflammation markers in the mouse
Source: PLoS One. 2024 Mar 21;19(3):e0300141. doi: 10.1371/journal.pone.0300141 (PMC10956830; doi:10.1371/journal.pone.0300141)
Supplement: S2 Fig — A) Three individual hypothalami from AA/SBO male and female experimental groups; original gels are shown in S1 and S2_raw_images in S1 Raw images. B) Pooled sample of RNA from 3 male and three female hypothalami i.e. samples shown in A; original gels are shown in S3_raw_image in S1 Raw images. (PDF) [file pone.0300141.s005.pdf]

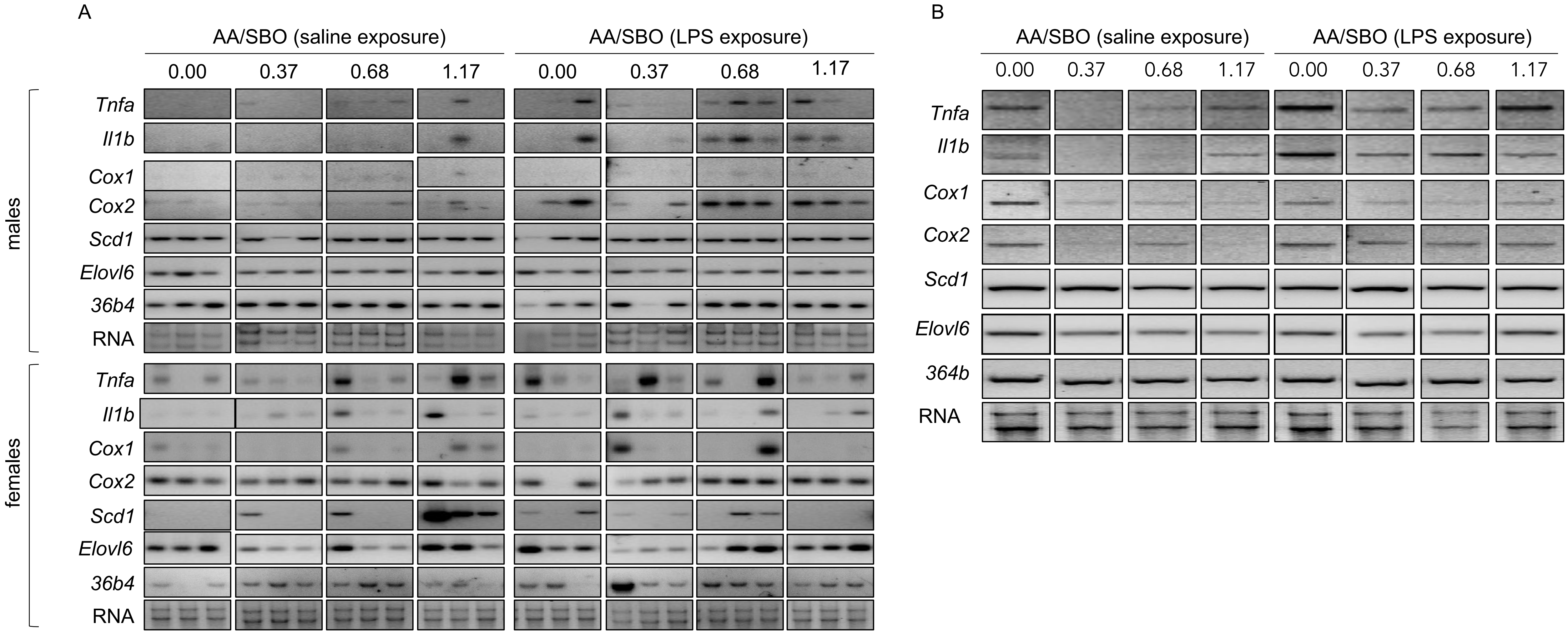

Supplemental Fig 2 - RT-PCR analysis of selected genes related to inflammation and fatty acid synthesis. A) Three individual hypothalami from AA/SBO male and female experimental groups**;** original gels are shown in S1-2_raw_image. B) Pooled sample of RNA from 3 male and three female hypothalami i.e. samples shown in A; original gels are shown in S3_raw_image.
