# Supplementary material for: Effects of paternal arachidonic acid supplementation on offspring behavior and hypothalamus inflammation markers in the mouse
Source: PLoS One. 2024 Mar 21;19(3):e0300141. doi: 10.1371/journal.pone.0300141 (PMC10956830; doi:10.1371/journal.pone.0300141)
Supplement: S3 Fig — Expression values of each gene were normalized to 36b4; f and m, female and male, respectively; asterisks indicate significance in post hoc analysis; *,***, *****, *****, p<0.1, 0.05, 0.01, 0.0001 and 0.00001, respectively; (n = 3/founder AA/SBO group). (PDF) [file pone.0300141.s006.pdf]

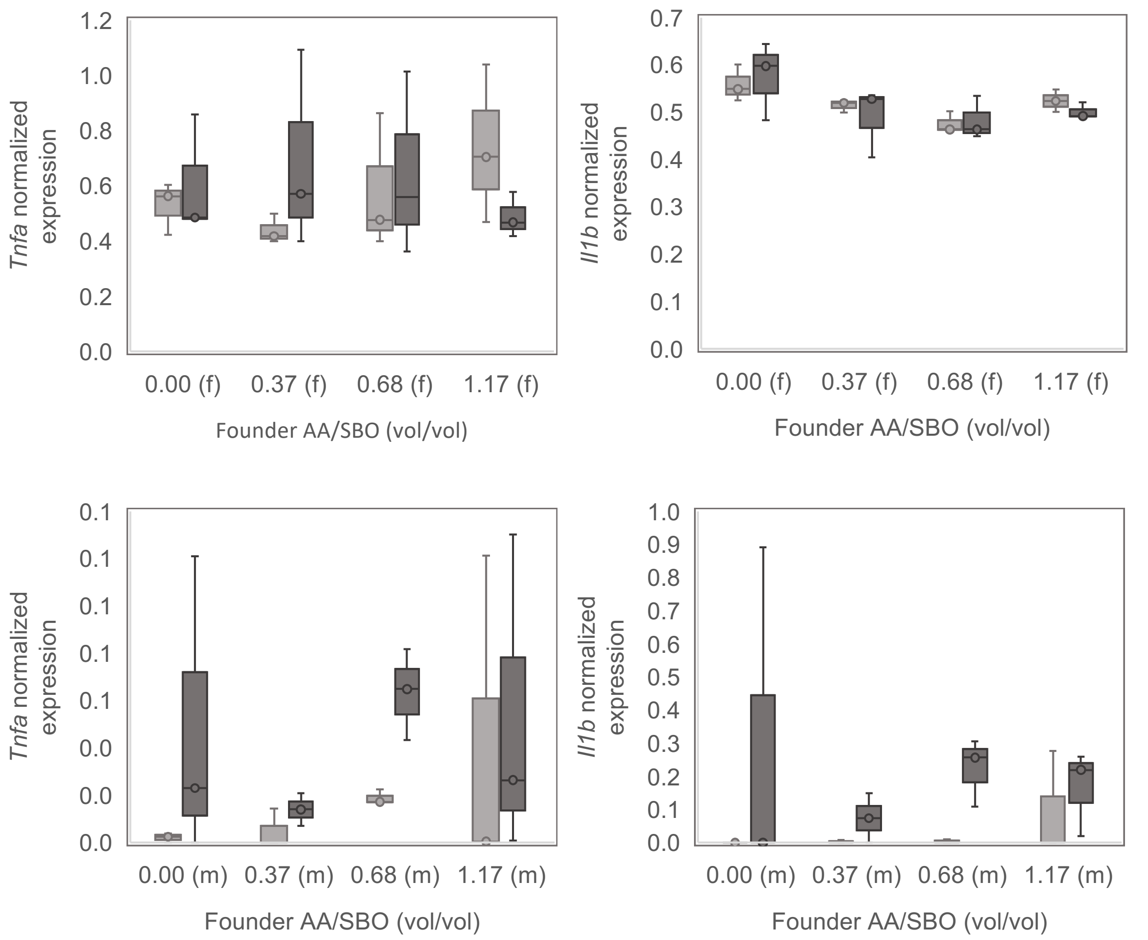

Supplemental Fig 3 - RT-PCR analysis of selected genes related to inflammation and fatty acid synthesis. Expression values of each gene were normalized to *36b4*; f and m, female and male, respectively; asterisks indicate significance in *post hoc* analysis; *,***, *****, *****, p<0.1, 0.05, 0.01, 0.0001 and 0.00001, respectively; (n=3/founder AA/SBO group).
