# Supplementary material for: Effects of paternal arachidonic acid supplementation on offspring behavior and hypothalamus inflammation markers in the mouse
Source: PLoS One. 2024 Mar 21;19(3):e0300141. doi: 10.1371/journal.pone.0300141 (PMC10956830; doi:10.1371/journal.pone.0300141)
Supplement: S1 Raw images — (PDF) [file pone.0300141.s007.pdf]

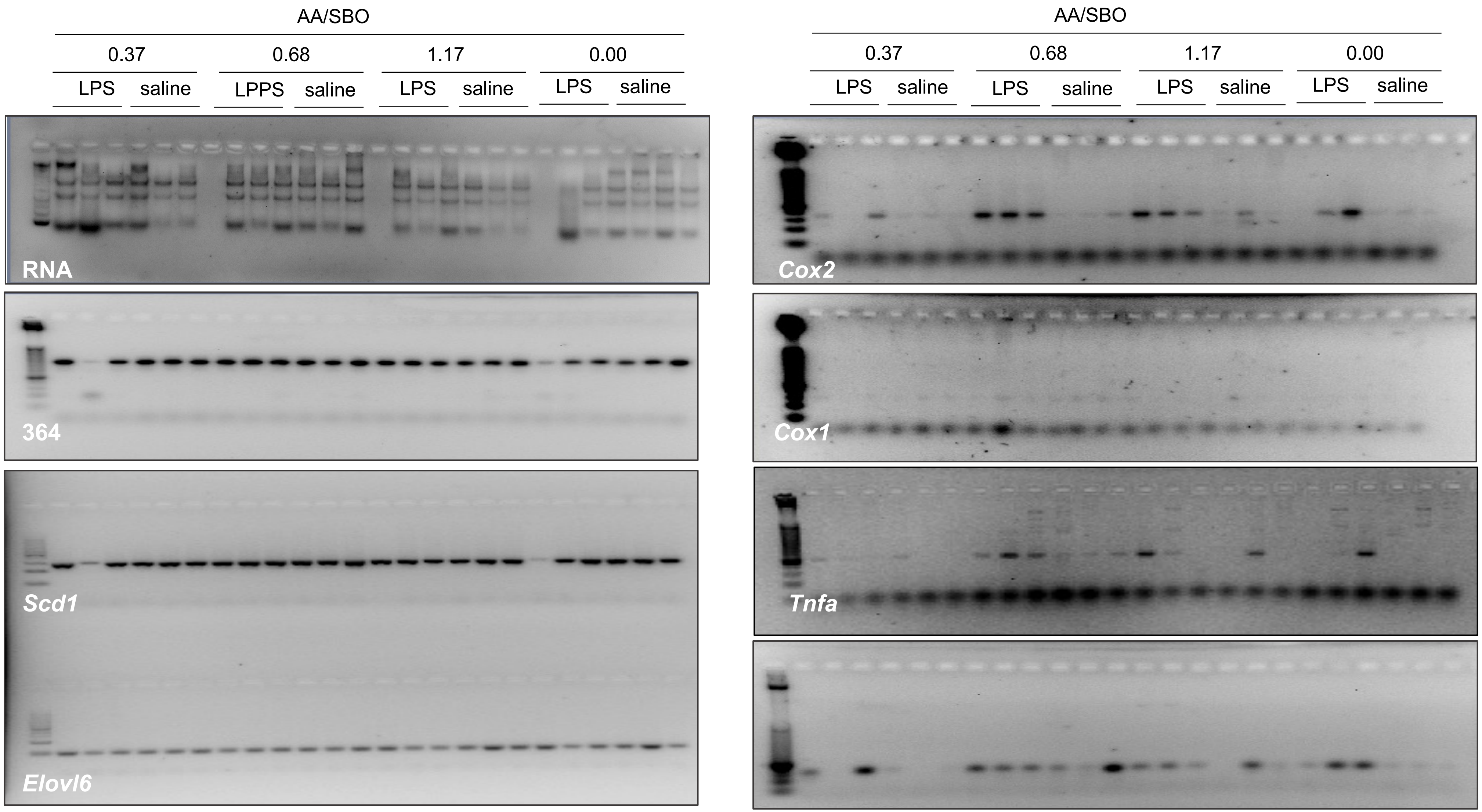

S1_raw_image – Uncropped versions of RT-PCR analysis shown in Supplemental Figure 2A (three male hypothalami of each AA/SBO group).

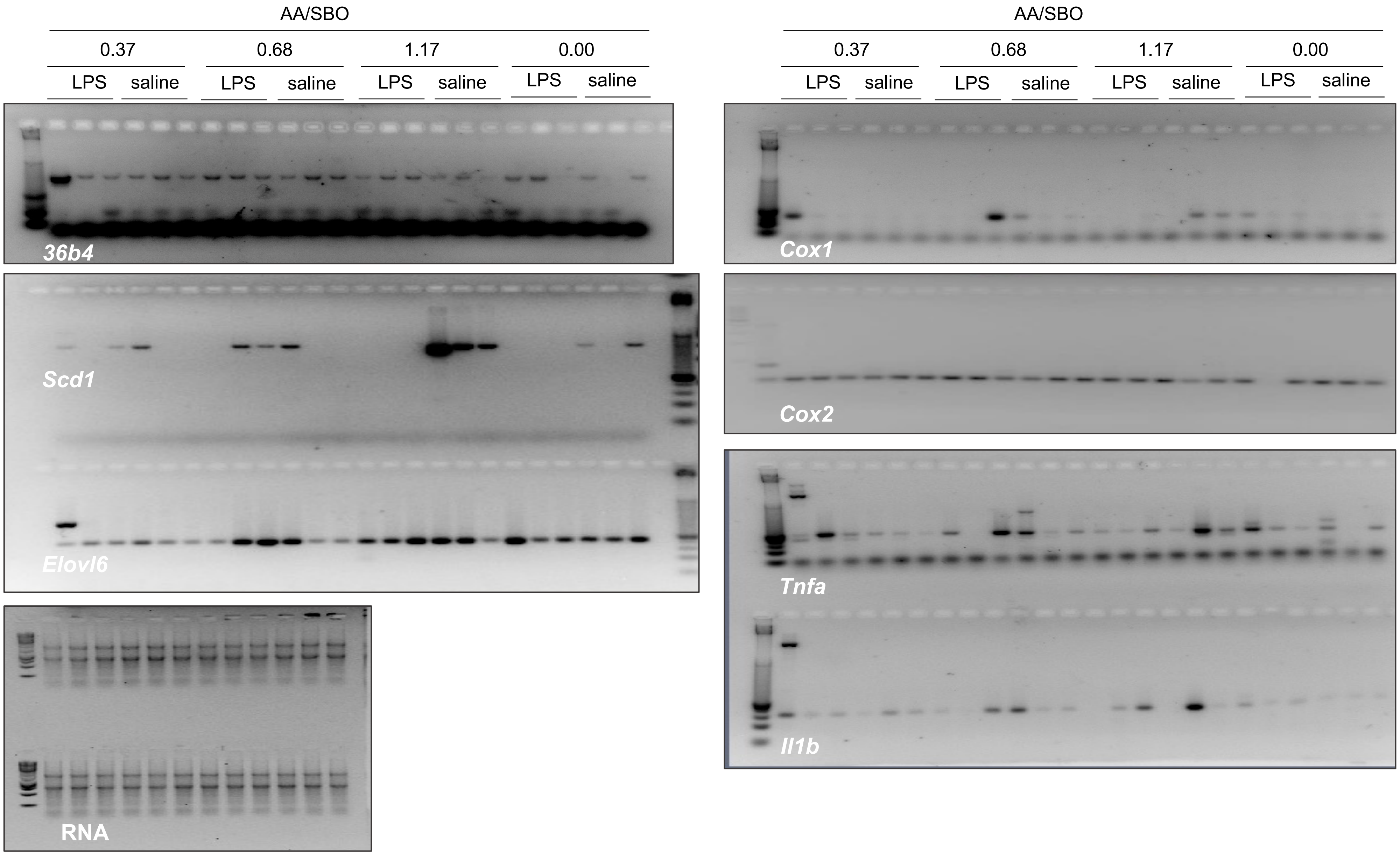

S2_raw_image – Uncropped versions of RT-PCR analysis shown in Supplemental Figure 2A (three female hypothalami of each AA/SBO group).

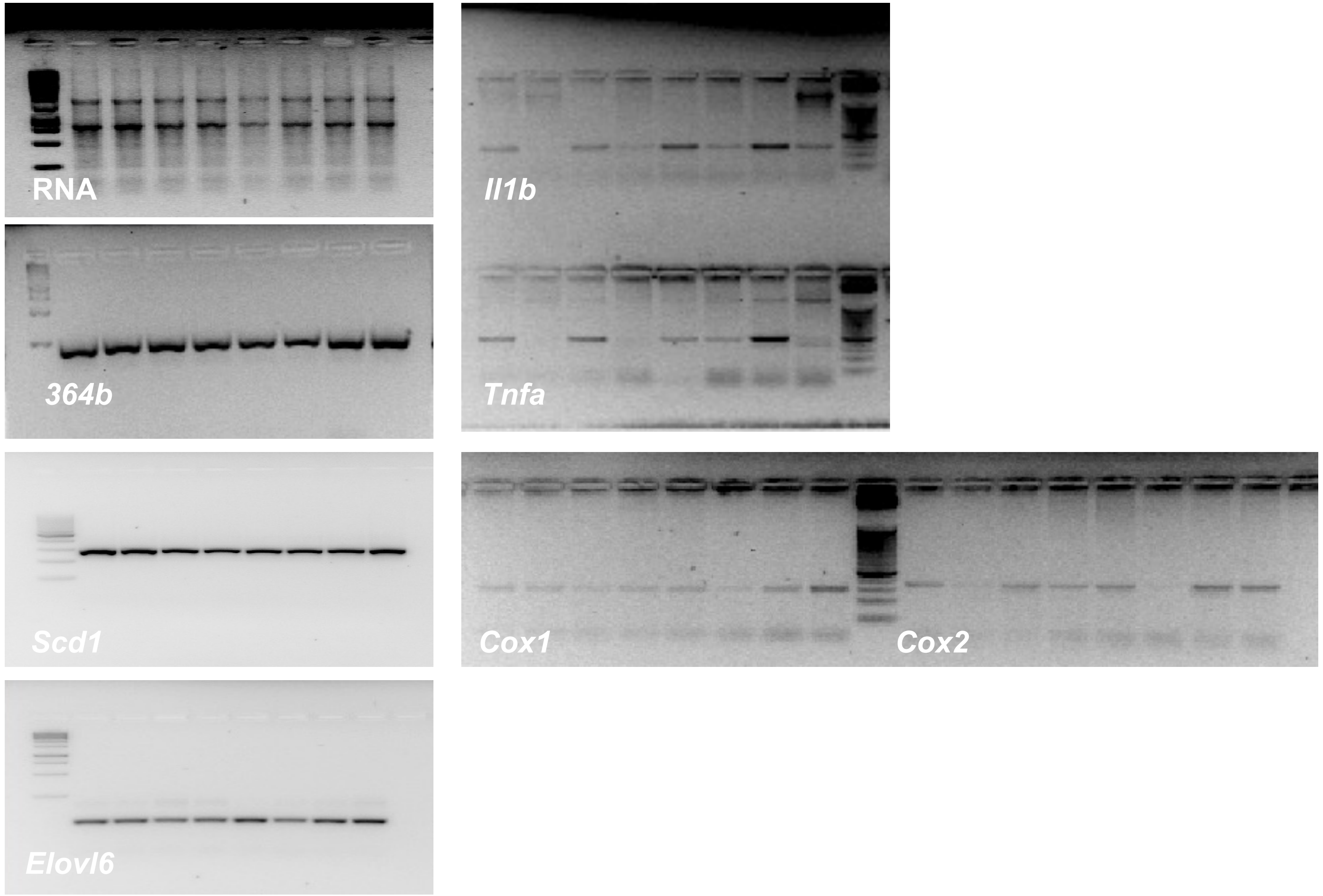

S3_raw_image – Uncropped versions of RT-PCR analysis shown in Supplemental Figure 2B (RNA pooled from 3 male and three female hypothalami).
